# Hierarchical Distribution of Reward Representation in the Cortical and Hippocampal Regions

**DOI:** 10.1523/ENEURO.0256-25.2026

**Published:** 2026-02-10

**Authors:** Shogo Soma, Masahiro Okamoto, Yui Mimura, Yoshikazu Isomura

**Affiliations:** ^1^Brain Science Institute, Tamagawa University, Tokyo 194-8610, Japan; ^2^Department of Molecular Cell Physiology, Kyoto Prefectural University of Medicine, Kyoto 602-8566, Japan; ^3^Department of Systems Neuroscience, Fukushima Medical University School of Medicine, Fukushima 960-1295, Japan; ^4^Department of Physiology and Cell Biology, Graduate School of Medical and Dental Sciences, Institute of Science Tokyo, Tokyo 113-8519, Japan

**Keywords:** association cortex, goal-directed behavior, hierarchical reward representation, hippocampal formation, interpretable machine learning, motor cortex

## Abstract

Dopaminergic inputs to various brain regions, such as the striatum, orbitofrontal cortex, and amygdala, play a critical role in processing reward acquisition information. While reward-related activity is also observed more broadly in motor, parietal, and hippocampal regions, the functional significance and potential hierarchy of reward-related representation across these latter areas remain unclear. We investigated this by quantifying neural predictive power using machine learning. Specifically, neural activity was examined in six brain areas—the primary and secondary motor cortices (M1 and M2), posterior parietal cortex (PPC), dorsal and ventral CA1 (dCA1 and vCA1), and lateral entorhinal cortex (LEC)—in male rats performing a self-initiated left–right choice task. Machine learning models classified rewarded versus nonrewarded trials based on neuronal firing properties significantly above chance for all regions. Crucially, classification revealed a clear performance gradient, forming a functional hierarchy: models using hippocampal data (dCA1 and vCA1) performed best, followed by LEC and PPC, with M1 and M2 performing lowest. Furthermore, SHapley Additive exPlanations (SHAP) analysis revealed a qualitative transformation in coding strategies along this hierarchy: while neocortical regions relied on subtle, distributed high-order statistics, the hippocampus utilized precise, categorical representations. At this apex, distinct strategies emerged: dCA1 primarily utilized temporally precise post-reward spike distributions with transient increase of response, while vCA1 integrated both spike timing and firing rate changes with suppressive response. These findings provide quantitative evidence for a functionally hierarchical and qualitative evolution of reward-related representation, highlighting distinct roles of dCA1 and vCA1 in encoding reward-related events to potentially guide future behavior.

## Significance Statement

How the brain represents reward information across distributed networks remains unclear. We used machine learning to quantitatively compare neural representations across six brain regions (M1, M2, PPC, LEC, dCA1, vCA1) in male rats performing a choice task. We identified a robust functional hierarchy: the hippocampus provided the most accurate reward prediction, significantly outperforming the motor cortices, with intermediate performance in the parahippocampal and parietal regions. Crucially, this hierarchy reflects a qualitative transformation from graded, distributed cortical codes to precise, categorical hippocampal representations. Furthermore, distinct coding strategies emerged at the hierarchy's apex: dCA1 relied on precise spike timing, while vCA1 integrated timing with firing suppression. This study reveals how reward information evolves across neural circuits to guide goal-directed behavior.

## Introduction

For survival, it is essential to process information about reward acquisition and use it to guide subsequent behavior in a goal-directed manner (Grace et al., 2007; Nonomura et al., 2018; Soma et al., 2018; Lee et al., 2021; Rios et al., 2025). Reward acquisition signals originating from dopamine neurons are transmitted to various brain regions and consist of three phases: fast, slow, and tonic components. These components are associated with distinct cognitive functions—prediction error, behavioral action and reward processing, and cognition/attention/motivation, respectively (Schultz, 2007, 2016).

The fast component has been observed in the midbrain dopamine system, specifically in the substantia nigra pars compacta (SNc) and ventral tegmental area (VTA; Schultz et al., 1997; Matsumoto and Hikosaka, 2009). In contrast, the slow and tonic components are distributed across brain regions that receive dopaminergic innervation (Berger et al., 1976; Garris and Wightman, 1994), such as the striatum (Young et al., 1992; Howe et al., 2013), orbitofrontal cortex (Pickens et al., 2003; Cetin et al., 2004; Izquierdo et al., 2004; Takahashi et al., 2011), and amygdala (Pickens et al., 2003; Paton et al., 2006), where they contribute to diverse neural processes.

Beyond these dopamine-governed regions, reward-related activity has also been observed in the motor, parietal, and hippocampal areas. For instance, in monkeys performing an arm-reaching task, neurons in the primary motor cortex (M1) and premotor cortex encode reward expectation and acquisition (Ramkumar et al., 2016; Ramakrishnan et al., 2017). Similarly, in freely moving or head-fixed rodents performing forelimb or tongue movements task, reward-related modulation has been reported in M1 and higher-order motor cortices, including the secondary motor cortex (M2) and the anterior–lateral motor cortex (Sul et al., 2011; Soma et al., 2017; Chen and Fontanini, 2025). Additional studies have identified reward signals in the parietal cortex (Sugrue et al., 2004), dorsal CA1 (dCA1; Gauthier and Tank, 2018; Soma et al., 2023; Issa et al., 2024; Sakairi et al., 2025), and lateral entorhinal cortex (LEC; Soma et al., 2023; Issa et al., 2024).

While reward-related activity is known to be widely distributed, how the quality and robustness of this information representation compare across brain regions remains poorly understood. Most studies have focused on one or two areas in isolation, making it difficult to directly assess the relative contribution of each region to reward processing. Therefore, a key unanswered question is whether a functional hierarchy exists in how robustly different brain areas encode reward-related information. To address this gap, it is necessary to systematically compare neural activity across multiple brain regions in animals performing the same reward-related task using identical methodologies. In recent years, machine learning has become indispensable in neuroscience to elucidate spatiotemporal information-encoding mechanisms (Bakhurin et al., 2017; Tunes et al., 2022; Mehrabbeik et al., 2023; Vivekanandhan et al., 2023). For example, it offers a comprehensive approach to explicitly quantify and contrast the efficacy of different areas in encoding information (Bakhurin et al., 2017). Moreover, machine learning techniques enable researchers to identify specific patterns in neural activity that respond to information representation (Mehrabbeik et al., 2023; Vivekanandhan et al., 2023). These methodologies represent a paradigm shift beyond cell identification methods, providing a comprehensive perspective on temporal representation as a dynamic evolution of population neural activity.

In this study, neural activity was recorded from six brain regions [M1, M2, posterior parietal cortex (PPC), dCA1, ventral CA1 (vCA1), and LEC] in rats engaged in a simple left–right choice task, where rewards were obtained through self-initiated behavioral choices without external cues. Using the firing properties of each region as feature variables, a machine learning model was constructed to classify trials as either successful or failed. This approach provided an objective framework not only to compare neural activity across regions but also to quantitatively assess the functional hierarchy of reward representation by using the model's classification performance.

## Materials and Methods

### Animals

All experiments were approved by the Animal Research Ethics Committee of Tamagawa University (animal experiment protocol, H22/27-32) and were carried out in accordance with the Fundamental Guidelines for Proper Conduct of Animal Experiment and Related Activities in Academic Research Institutions (Ministry of Education, Culture, Sports, Science, and Technology of Japan) and the Guidelines for Animal Experimentation in Neuroscience (Japan Neuroscience Society). All surgical procedures were performed under appropriate isoflurane anesthesia (see below). All effort was made to minimize suffering. The procedures for the animal experiments were established in previous studies and are described in detail below (Soma et al., 2017; Rios et al., 2019). This study is based on data from channelrhodopsin-2 (ChR2)–expressing (Thy1-ChR2) transgenic rats (W-TChR2V4; *N* = 33 rats; male; >3 months) abundantly expressing ChR2-Venus fusion protein under the control of the *Thy1.2* promoter in cortical and other neurons (Tomita et al., 2009; Saiki et al., 2018). These animals were kept in their home cage under an inverted light schedule (lights off at 9 A.M., lights on at 9 P.M.).

### Surgery

Rats were handled briefly by the experimenter (10 min, twice) before the day of surgery. For head plate implantation, rats were anesthetized with isoflurane (4.5% for induction and 2.0–2.5% for maintenance; Pfizer) using an inhalation anesthesia apparatus (Univentor 400 anesthesia unit, Univentor) and placed on a stereotaxic frame (SR-10R-HT, Narishige). In addition, lidocaine jelly (AstraZeneca) was administered around surgical incisions for local anesthesia. During anesthesia, body temperature was maintained at 37°C using an animal warmer (BWT-100, Bio Research Center). The head plate (CFR-2, Narishige) was attached to the skull with small anchor screws and two combination of dental resin cements (Super-Bond C&B, Sun Medical; Unifast II, GC Corporation). Reference and ground electrodes (Teflon-coated silver wires, A-M Systems; 125 µm in diameter) were implanted above the cerebellum. Analgesics and antibiotics were applied after the operation (meloxicam, 1 mg/kg, s.c., Boehringer Ingelheim; gentamicin ointment, 0.1% ad usum externum, Merck Sharp & Dohme).

Water deprivation was started after full recovery from surgery (6 d postoperatively). The rats had *ad libitum* access to water during weekends, but during the rest of the week, they obtained water only by performing the task correctly. When necessary, an agar block (containing 15 ml water) was given to the rats in their home cage to maintain them at >85% of their original body weight (Soma et al., 2021; Suematsu et al., 2025).

### Behavioral task

**Figure 1.**
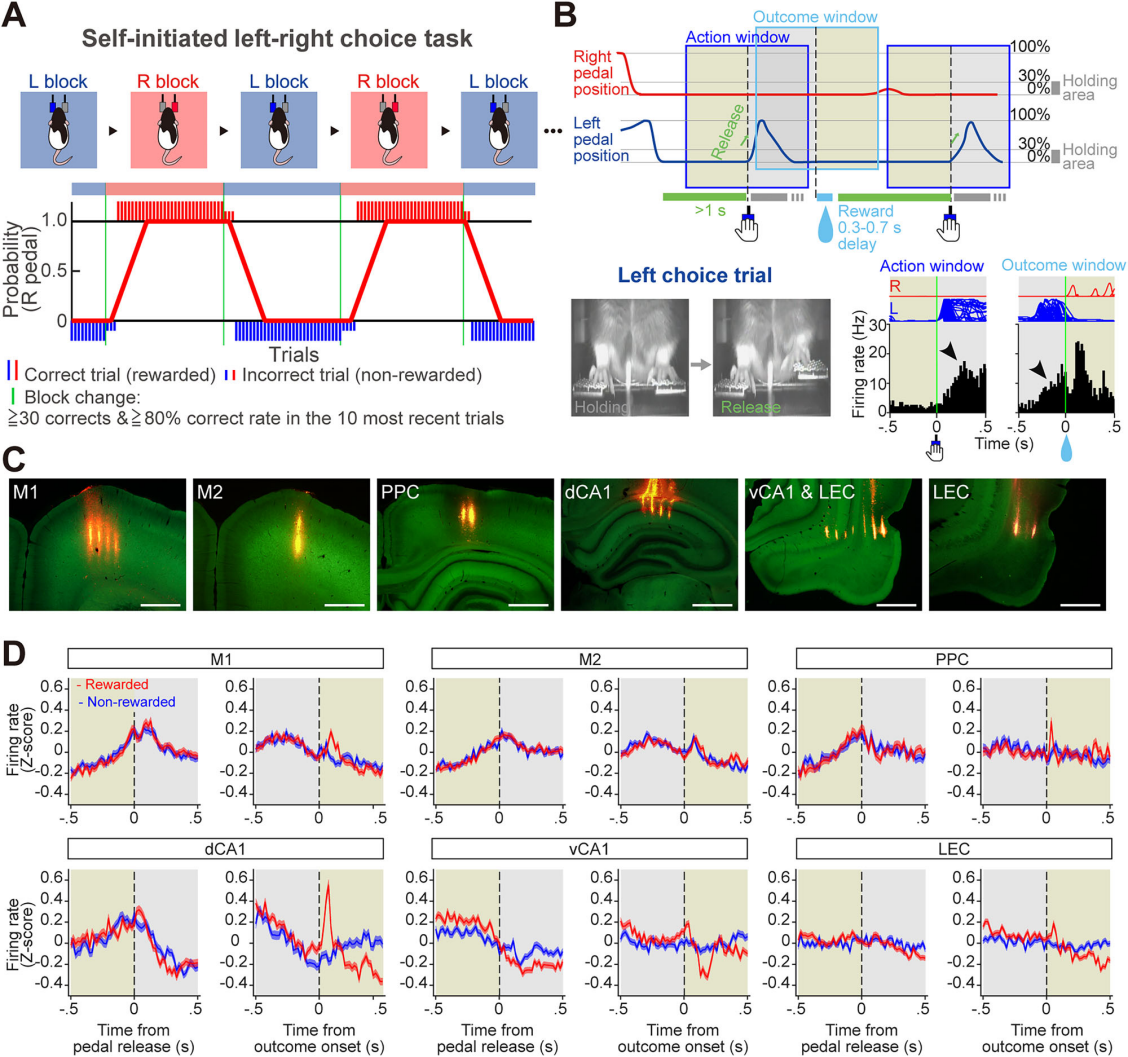
Electrophysiological recordings of neuronal activity during a self-initiated left–right choice task. ***A***, ***B***, Schematic diagrams of the behavioral task (***A***, top; ***B***, top) and examples of task performance (***A***, bottom) and task-related activity (***B***, bottom). Head-fixed rats depressed both pedals for ≥1 s to initiate each trial and then voluntarily released either pedal (e.g., left release) without any external cue to obtain a reward. The reward was delivered after a random delay of 0.3–0.7 s. The task consisted of right-rewarded (R) and left-rewarded (L) blocks, which alternated once a performance criterion was met (***A***, see Materials and Methods). The rat selected the correct pedal based on the current reward contingency. Large- and small-colored vertical bars (red, right choice; blue, left choice) represent correct and incorrect trials, respectively. The proportion of correct choices was calculated as the average number of right-correct choices over the previous 10 trials. Example of task-related activity recorded from the LEC (***B***, bottom). PETHs were constructed using pedal release and outcome onset as trigger points. We analyzed the pre-action and post-outcome windows (pale yellow areas) but excluded the post-action and pre-outcome windows (gray areas) because they contained movement-related activity (arrowhead). ***C***, Recording sites in the primary (M1) and secondary (M2) motor cortices, the PPC, dCA1 and vCA1, and LEC. Probe shank tracks were visualized by fluorescent DiI (red). ***D***, Averaged PETHs of all recorded neurons among six brain areas. PETHs were aligned with pedal release onset (left) and reward onset at 0 s (right). Shaded regions represent ±SEM.

A self-initiated left–right choice task (the LR pedal task) was used in the original system (custom made by O'HARA; Fig. 1*A*,*B*) to examine the timing of neural representation of two distinct behavioral events related to action and outcome in six brain regions (M1, M2, PPC, dCA1, vCA1, and LEC). In this task, the rats had to manipulate left and right pedals with the corresponding forelimb in a head-fixed condition. They spontaneously started each trial by pushing both pedals down with both forelimbs and holding them down for a short period (“holding period,” at least 1 s; Fig. 1*B*). After completing the holding period, the rats had to release either the left or the right pedal, depending on the context without any instruction cue, to obtain 0.1% saccharin water (10 µl) as a reward. The reward was dispensed from the tip of a spout by a micropump with a 0.3–0.7 s delay (0.1 s steps at random). If the rats incorrectly released the other pedal (nonrewarded trial), then they were given an error sound and were not rewarded (3 kHz, 0.3 ms). If they did not complete the holding period (immature trial), then they did not receive feedback. This task consisted of two blocks, right pedal-rewarded and left pedal-rewarded blocks. Each block lasted until the rat performed >30 correct (rewarded) trials and achieved 80% correct performance in the 10 most recent trials or until 100 rewards had been obtained (Fig. 1*A*). The rats typically learned this task within 2 weeks (2–3 h per day).

Once the rats completed task learning, they underwent a second surgery under isoflurane anesthesia for later recording experiments. Tiny holes (1.0–1.5 mm in diameter) were made in the skull and dura mater above the vCA1 (5.0 mm posterior, 5.0 mm lateral from the bregma), the dCA1 (4.5 mm posterior, 2.0 mm lateral from the bregma), the LEC (6.0 mm posterior, 6.8 mm lateral from the bregma), the PPC (3.8 mm posterior, 2.4 mm lateral from the bregma), the M2 (3.5 mm anterior, 2.4 mm lateral from the bregma), and the M1 (1.0 mm anterior, 2.5 mm lateral from the bregma). All holes were immediately covered with silicone sealant (DentSilicone-V, Shofu) until the recording experiments.

### In vivo electrophysiological recording

Extracellular multineuronal (multiple, isolated, single-unit) recordings from individual neurons were performed while the rats were performing behavioral tasks. A supportive layer of agarose gel (2% agarose-HGT, NACALAI TESQUE) was placed on the brain, and then 32-channel silicon probes (Iso_3x_tet-A32 or Iso_4x_tet-A32; NeuroNexus Technologies) were precisely inserted into one or two of six targeted brain regions. Insertions were performed using fine micromanipulators (SM-15 or SMM-200B, Narishige) at least 1 h before the start of each recording experiment.

Wide-band signals were amplified and filtered (FA64I, Multi Channel Systems; final gain, 2,000; bandpass filter; 0.5 Hz–10 kHz) through a 32-channel head stage (MPA32I, Multi Channel Systems; gain, 10). These signals were digitized at 20 kHz and recorded with three 32-channel hard-disc recorders (LX-120, TEAC) that simultaneously digitized the pedal positions tracked by angle encoders.

### Spike isolation

Wide-band signal data were processed offline to isolate spike events of individual neurons in each tetrode of the silicon probes. Briefly, wide-band signals were bandpass filtered (800 Hz–3 kHz) for spike detection, and spike candidates were detected and clustered by a semiautomatic spike-sorting software, EToS (Takekawa et al., 2010, 2012). Using open-source software (Klusters clustering software and NeuroScope viewing software; Hazan et al., 2006), spike clusters were further combined, divided, and/or discarded manually to refine single-neuron clusters based on the presence of refractory periods (>2 ms) in their own autocorrelograms (ACG) and from the absence of refractory periods in cross-correlograms with other clusters. Single-neuron clusters were included for further analysis.

### Spike analysis

**Figure 2.**
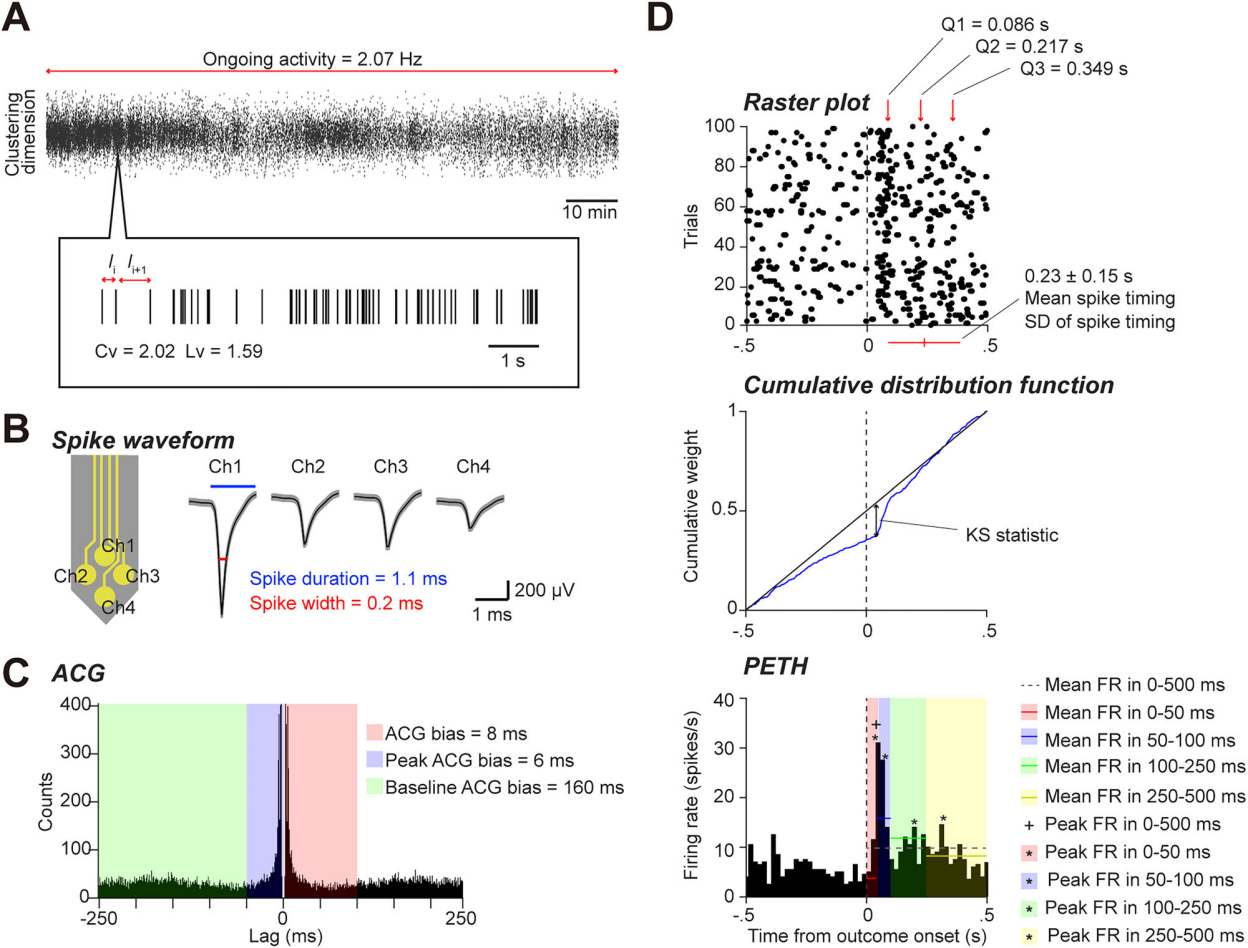
Illustration of representative features. ***A***, Spike time events are shown along one of the clustering dimensions. Ongoing activity was defined as the grand average of spiking activity across the entire recording period. Basic temporal features of ISIs ( $I_{i}$, $I_{i+1}$) were quantified using the conventional coefficient of variation (Cv) and local variation (Lv). ***B***, Spike waveform features were characterized by spike duration and spike width, calculated from the spike waveform with the maximum amplitude recorded on each tetrode. ***C***, Temporal features from the ACG included the classical ACG bias, defined as the median value within 0–100 ms. Spike burstiness and postspike suppression were quantified using peak ACG bias (median at 0–50 ms) and baseline ACG bias (median at 50–250 ms). ***D***, Task-related spike–timing properties were derived from raster plots (top) and their cumulative distribution functions (middle). Task-related firing rate features were calculated from PETHs (bottom). See Materials and Methods and Extended Data Figure 2-1 for further details.

For each neuron (spike cluster), basal spiking properties and neuronal dynamics related to behavioral task performance were analyzed using MATLAB (MathWorks; Fig. 2). The ongoing spike rate, spike duration, and spike width for individual spike clusters were defined following the previous studies (Isomura et al., 2009). Briefly, the spike duration was the time from spike onset to the first positive peak, and the spike width referred to the elapsed time above the half amplitude of the positive spike waveform.

A temporal feature in its ACG was evaluated by defining classical “ACG bias” as the median value in a time-window from 0 to +100 ms in ACG (Saiki et al., 2014). Spike burstiness and postspike suppression were assessed by two additional indexes (Saiki et al., 2018), peak ACG bias (median at 0–50 ms) and baseline ACG bias (median at 50–250 ms).

Other temporal features of spike, interspike interval (ISI), were evaluated using the conventional coefficient of variation (Cv) and local variation (Lv) as described as follows (Shinomoto et al., 2009):

$$Cv\;=\frac{\mathrm{SD}\;\mathrm{of}\;\mathrm{ISIs}}{\mathrm{Mean}\;\mathrm{ISIs}},$$

where ISI is the interspike interval and SD represents the standard deviation.

$$Lv\;=\;\frac{3}{n-1}\overset{n-1}{\underset{i\;=\;1}{\sum }}\;\left(\frac{I_{i}-I_{i+1}}{I_{i}+I_{i+1}}\right),$$

where $I_{i}$ and $I_{i}+1$ are the *i*th and *i* + 1 ISIs and *n* is the number of ISIs. Both Cv and Lv adopt a value of 0 for a sequence of perfectly regular intervals and are expected to take a value of 1 for a Poisson random series of events with ISIs that are independently exponentially distributed.

Next, a perievent time histogram (PETH) was constructed to examine neuronal dynamics associated with self-initiated actions (pedal-releasing) and outcomes (reward delivery/error sound). For action-related activity, spike trains were analyzed in relation to unilateral forelimb movements during task performance (contralateral and ipsilateral trials), aligning them to the onset (0 s) of pedal release (after ≥1 s of holding; window, onset ±500 ms). The pedal's range of motion was 0–100%, with the holding area defined as 0–30% (Fig. 1*B*). Task progression was marked by pedal release, detected when the pedal moved beyond the holding area. To more precisely determine release onset for neuronal analysis, pedal release was defined as the moment the pedal position exceeded 5% before reaching full release (compared with the standard 30% detection threshold; see above). For reward-related activity, spike trains were aligned to the onset of the reward delivery (0 s; window, onset ±500 ms). For nonrewarded trials, this was the onset of the auditory error cue, as this cue signaled the trial's outcome to the animal. To distinguish trial types, they were referred to as action-contralateral (AC), action-ipsilateral (AI), outcome-contralateral (OC), and outcome-ipsilateral (OI).

To assess changes in spike distribution related to action or outcome, a Kolmogorov–Smirnov (KS) test was performed, as previously described (Saiki et al., 2014; Soma et al., 2017). The cumulative distribution of all spike positions within each trial was compared with a uniformly distributed set of spike positions of the same number. The KS statistic was calculated separately for rewarded and nonrewarded trials.

The firing rate change (FRc) index, which quantifies changes in action- and outcome-related activity, was calculated using the following equation:

$$\mathrm{FRc}\;\mathrm{index}=(FR_{\mathrm{post}}-FR_{\mathrm{pre}})/(FR_{\mathrm{post}}+FR_{\mathrm{pre}}),$$

where FR_pre_ is the mean firing rate (mean FR) during the period before the action or outcome (−500 to 0 ms) and FR_post_ is the mean FR during the period after the action or outcome (0–500 ms). This index was calculated separately for rewarded and nonrewarded trials. A positive FRc index (>0) indicates that neural activity is positively modulated by action or outcome.

### Histological observations

After the recording experiments, animals were deeply anesthetized with urethane (2–3 g/kg, i.p.; NACALAI TESQUE) and transcardially perfused with cold saline followed by 4% formaldehyde in 0.1 M phosphate buffer. Whole brains were postfixed and sliced coronally into 50 µm serial sections using a microslicer (VT1000S, Leica). Electrode tracks labeled with 1,1′-dioctadecyl-3,3,3′,3′-tetramethylindocarbocyanine perchlorate (DiI, Thermo Fisher Scientific) were observed in six brain regions under a fluorescence microscope (BX51N, Olympus).

### Dataset

The final dataset for this study comprised electrophysiological data from 71 recording sessions conducted in a total of 33 Thy1-ChR2 male rats. This dataset was used to examine reward-related representations across six brain regions. This comprehensive dataset was compiled by combining newly acquired data with previously published datasets (Soma et al., 2017, 2019, 2023), which is justified by the use of identical experimental setups, behavioral tasks, recording methods, and spike-sorting criteria across all experiments. The detailed breakdown of sessions and animals contributing to each region is as follows: M1, 23 sessions/16 rats, mean ± SD, 1,064 neurons, 35 ± 25 neurons/session; M2, 28 sessions/15 rats, 1,463 neurons, 44 ± 26 neurons/session; PPC, 13 sessions/8 rats, 694 neurons, 43 ± 28 neurons/session; dCA1, 12 sessions/11 rats, 744 neurons, 37 ± 29 neurons/session; vCA1, 7 sessions/5 rats, 1,096 neurons, 157 ± 45 neurons/session; and LEC, 15 sessions/15 rats, 1,619 neurons, 70 ± 107 neurons/session. It is important to note that the total number of unique animals (33) is less than the sum of rats per region because some animals contributed data from multiple regions or participated in multiple recording sessions (single-session/day). For instance, recordings were taken from one to three brain regions per day from a single animal. To construct the feature matrix, we first filtered at the trial level: we used all trials except those in which rats moved both forelimbs during the post-action period. In other words, only action trials that were clearly defined as ipsilateral or contralateral movements were included. The detailed breakdown of sample numbers and further experimental conditions is summarized in Table 1.

**Table 1.** Dataset statistics and classification performance of the hyperparameter-tuned models

			M1	M2	PPC	LEC	vCA1	dCA1
Sample size		Reward	915	1,277	661	1,298	1,028	610
		Nonreward	891	1,221	629	1,164	750	518
		Total	1,806	2,498	1,290	2,462	1,778	1,128
Tuned models and performance on test data	CatBoost	Accuracy (SD)	0.6408 (0.0248)	0.6524 (0.0235)	0.6933 (0.0403)	0.7704 (0.0090)	0.8027 (0.0416)	0.8083 (0.0230)
		AUC (SD)	0.6940 (0.0352)	0.7038 (0.0234)	0.7597 (0.0331)	0.8472 (0.0085)	0.8828 (0.0385)	0.8928 (0.0217)
	LightGBM	Accuracy (SD)	0.6390 (0.0102)	0.6569 (0.0239)	0.6684 (0.0368)	0.7618 (0.0160)	0.8121 (0.0319)	0.7935 (0.0223)
		AUC (SD)	0.6824 (0.0341)	0.7062 (0.0280)	0.7386 (0.0414)	0.8359 (0.0206)	0.8809 (0.0322)	0.8871 (0.0180)
	XGBoost	Accuracy (SD)	0.6310 (0.0394)	0.6369 (0.0245)	0.6821 (0.0506)	0.7573 (0.0082)	0.7984 (0.0309)	0.7994 (0.0340)
		AUC (SD)	0.6792 (0.0445)	0.6957 (0.0190)	0.7440 (0.0511)	0.8397 (0.0106)	0.8787 (0.0309)	0.8902 (0.0196)

The table summarizes the number of samples analyzed and the classification performance of the best-performing machine learning models (CatBoost; LightGBM, Light Gradient Boosting Machine; XGBoost, Extreme Gradient Boosting) selected for each brain region (M1, M2, PPC, LEC, vCA1, dCA1). Sample size represents the total number of valid neuron-condition data points used for classification (see Materials and Methods). Performance Metrics, accuracy and AUC values, represent the mean and standard deviation (SD) calculated across three independent repetitions of the hyperparameter tuning and evaluation process. Note that the hippocampal regions (dCA1, vCA1) consistently achieved the highest performance metrics with low variance, confirming the robustness of the functional hierarchy.

### Machine learning classification

To determine whether each of the six domains retains information relevant to the reward task, we employed a “neuron-by-neuron” classification approach based on the dataset described above. The analysis was constructed from the spiking dynamics of individual neurons, treating each neuron as an independent statistical sample. Specifically, for the binary classification task, two distinct data points (i.e., rows in our feature matrix) were created from each neuron: one representing its averaged activity profile under the “rewarded” condition and one for the “nonrewarded” condition. Theoretically, this would result in a total sample size exactly twice the number of recorded units. However, if a neuron lacked sufficient trial data for robust feature calculation under a given condition, that data point was excluded. Therefore, the final, actual sample size used for classification, which reflects these exclusions, is reported in Table 1.

This “neuron-by-neuron” approach was intentionally chosen for two main reasons. First, from a scientific perspective, it allows us to investigate the general, nontransient firing properties (e.g., firing rate vs spike timing patterns) that characterize a typical neuron's response to reward within each brain region's population. This stands in contrast to standard population decoding strategies, which typically rely on simultaneously recorded ensembles to capture information encoded in neuronal correlations and synergistic network interactions. By treating neurons as isolated samples, our analysis specifically targeted the intrinsic information capacity of the average neuron in each region, rather than momentary collective states.

Second, from a technical and practical perspective, this approach addresses critical constraints related to trial availability and data sparsity. Specifically, due to the high behavioral performance of the rats, the number of nonrewarded (error) trials was often limited within individual sessions. This scarcity made standard simultaneous population decoding—which requires sufficient trial numbers for both conditions within each session for robust cross-validation—impractical for many sessions. By pooling neurons across sessions, we could maximize the utility of the dataset. Furthermore, this averaging approach ensures that complex temporal features (e.g., Cv, spike timing skewness) can be robustly calculated, as detailed in the feature vector construction section below. A “trial-by-trial” analysis, in contrast, would suffer from sparse firing in short time windows, leading to numerous missing values (NAs) for these critical features. The limitations of this approach and the complementary insights offered by a “trial-by-trial” analysis are discussed further in the Discussion section.

Following this data construction, binary classification models were developed and evaluated. All machine learning analyses were performed on a host computer running Windows 11 Pro. To ensure a consistent and reproducible computational environment, we executed all analyses within a Docker container. The container environment was built from a python:3.10-slim (Debian-based) image and included key libraries such as PyCaret (Ali, 2020) for model training and SHapley Additive exPlanations (SHAP; Lundberg and Lee, 2017) for model interpretation. The complete environment is defined in the Dockerfile available in the code repository. All steps described below, from data preprocessing to model comparison, model tuning, and model interpretation, were performed using PyCaret (Ali, 2020).

### Feature vector construction

The feature vector for each data point (i.e., for each neuron's rewarded or nonrewarded condition, as defined in the machine learning classification section above) was composed of a wide array of 88 features (Fig. 2, Extended Data Fig. 2-1), which can be broadly categorized into three main groups for analysis.

The first group, “fundamental spiking properties,” includes features characterizing the intrinsic firing patterns and waveform of each neuron, independent of specific task events (Fig. 2, Extended Data Fig. 2-1). These task-independent features were included based on the hypothesis that a neuron's intrinsic properties could be predictive of its role in reward-related processing. For example, it is well known that interneurons often exhibit narrower spike widths and shorter ISIs compared with pyramidal cells (Isomura et al., 2009). A model could potentially leverage such combinations of intrinsic features and task-dependent activity to learn that a specific cell type is more likely to be involved in encoding reward-related information. These properties, calculated once per neuron, comprised measures of the action potential waveform (spike duration and spike width), ISI variability (Cv and Lv) used to assess firing regularity, and features from the ACG function (namely, classical ACG bias, peak ACG bias, and baseline ACG bias) used to assess temporal firing patterns like bursting or refractoriness. This provided the model with a comprehensive baseline characterization of each neuron, which was treated as constant across rewarded and nonrewarded conditions in this analysis.

The second group, “task-related spike–timing properties,” focuses on the precise timing and distribution of spikes relative to action and outcome triggers, derived from timestamp data within the ±500 ms analysis window. This category includes moments of the spike timing distribution such as the mean spike timing, SD of spike timing, skewness (asymmetry), and kurtosis (tailedness). It also incorporates the time points corresponding to the first (Q1, 25%), second (Q2, 50%, median), and third (Q3, 75%) quartiles of the spike time distribution. In addition, the KS statistic was calculated to quantify the difference between the observed cumulative distribution of spike times and a uniform distribution, assessing nonuniformity in spike timing. These timing properties were computed separately for contralateral (AC, OC) and ipsilateral (AI, OI) trials relative to the action or outcome trigger (Fig. 2, Extended Data Fig. 2-1).

The third group consists of “task-related firing-rate–based properties,” which were derived from PETHs aligned to action onset and outcome onset. These features quantify the magnitude and change in the firing rate around these task events. They included the mean FR calculated within various time windows relative to the trigger (e.g., 0–50 ms, 50–100 ms, 100–250 ms), the peak firing rate (Peak FR) observed within specific time windows, and the FRc index quantifying the relative change in firing rate between the 500 ms periods before and after the trigger (Eq. 3). Similar to the spike timing properties, these rate-based features were calculated separately for contralateral (AC, OC) and ipsilateral (AI, OI) trials relative to the action or outcome trigger, respectively (see Fig. 2 and Extended Data Fig. 2-1 for all specific windows and trial types).

The final, comprehensive feature vector for each data point was a concatenation of all the properties described above. This included task-dependent features derived from all combinations of epoch (action vs outcome) and movement direction (ipsilateral vs contralateral), i.e., AC, AI, OC, and OI conditions. This approach, rather than building separate decoders for each specific condition, allowed a single classifier to leverage all available information simultaneously—both the neuron's intrinsic properties and its diverse task-modulated responses—to find the most predictive patterns for the overall trial outcome.

To focus exclusively on neural information related to reward processing, we did not analyze activity during periods likely to be strongly influenced by forelimb movements (actions)—specifically, the post-action and pre-outcome epoch. This period includes neuronal activity immediately following pedal pressing and immediately preceding outcome delivery, which may primarily reflect the physical act of movement rather than reward-related or predictive processing (Fig. 1*B*). Therefore, these time windows were excluded from the final feature set used for modeling except for the calculation of the FRc (see above).

### Data preprocessing

Following the data preparation phase, a series of analytical procedures were conducted using PyCaret (Ali, 2020). These procedures included preprocessing, model comparison, model training (focusing on the top three models), hyperparameter tuning (for the top three models), and model evaluation.

The dataset underwent several preprocessing steps as follows: missing value imputation was conducted by imputing missing values with the mean. Feature scaling was performed using standard scaling, specifically *z*-score normalization. Class imbalance was addressed through the application of synthetic minority oversampling technique (SMOTE), which generates new synthetic samples by selecting examples that are proximate in the feature space and interpolating between them. Multicollinearity was assessed by examining the absolute Pearson's correlation coefficient between any two features; if this coefficient exceeded 0.80, the features were considered highly collinear. In such cases, the feature with the lower correlation to the response variable was removed. Feature selection was implemented to reduce dimensionality, thereby minimizing overfitting and enhancing computational efficiency. An automatic feature selection process was employed, which involved the following: automatic evaluation of all features based on their importance to the target variable. A Light Gradient Boosting Machine (LightGBM) model was trained on the preprocessed data to compute feature importance scores, determined by the frequency and effectiveness of features in decision splits. LightGBM is recognized for its rapid training and high accuracy, particularly with structured data, making it a robust choice for evaluating feature importance. It is capable of capturing complex, nonlinear relationships, ensuring the retention of the most informative features. The importance scores generated by LightGBM were subsequently used to rank the features. Only the top 20% of features, as ranked by their estimated importance, were retained for model training. These selected features were then utilized in subsequent steps, including model comparison, training, tuning, and testing.

### Model training and comparison

After the preprocessing and the preparation of the training data (80% of the full dataset), the performance of numerous classification algorithms was compared. These encompassed gradient boosting machines [CatBoost, Extreme Gradient Boosting (XGBoost), LightGBM], other ensemble methods [adaptive boosting (AdaBoost), decision tree, extra trees, random forest, gradient boosting], linear and discriminant models [linear discriminant analysis (LDA), logistic regression, quadratic discriminant analysis, ridge, support vector machine (SVM)], *K*-nearest neighbors, naive Bayes, and a dummy baseline. Each model was evaluated using a stratified 10-fold cross-validation strategy, ensuring performance was robust and generalizable. For each candidate model, key performance metrics [e.g., accuracy, F1 score, the area under the receiver operating characteristic curve (AUC), etc.] were computed during cross-validation. The results were averaged across the validation folds for each model and the models were ranked based on their performance (i.e., accuracy). A subset of models was identified for further tuning and analysis for each brain area. The candidate models were chosen based on the mean accuracy (the proportion of correctly predicted instances).

### Tune model

Subsequent tuning was conducted on the subset of models previously identified. This phase involved refining hyperparameters to further optimize the models. The tuned models were subsequently reevaluated. The procedure was executed as follows: a range of hyperparameters was assessed using stratified fivefold cross-validation to determine the optimal configurations that enhance model performance based on accuracy. This process was iterated for a predetermined number of iterations (i.e., 20), sampling various hyperparameter combinations. Optuna, an open-source library for hyperparameter optimization, was employed to explore the hyperparameter space. During this iterative search, the function compared performance metrics to select the optimal set of hyperparameter values. Upon completion of the tuning process, a new model object with the best-found hyperparameters was returned. This tuned model is anticipated to deliver superior performance compared with the original model with default parameters. In the event that the tuning process does not yield improvements, the original model was retained, ensuring the selection of the best-performing model for the task. To ensure robustness, this tuning procedure was repeated three times for each model and region combination.

### The tuned model evaluation

To thoroughly assess the generalization performance of the tuned models, we employed the held-out test set, comprising 20% of the total dataset, which was completely isolated from the training and tuning phases. For each brain region, the model with the optimal hyperparameters identified during tuning was retrained on the entire training set and subsequently evaluated on the test set. Importantly, to ensure the robustness of our findings and account for potential variability due to data splitting and model initialization, this entire evaluation process—from the train–test split to hyperparameter tuning and final testing—was repeated three times with different random seeds. The final performance metrics, specifically classification accuracy and the AUC, were calculated as the mean and SD across these three independent repetitions. These aggregated results reflect the model's capability to classify unseen neural data.

### Statistical verification of model performance: shuffle control analysis and ANOVA

To rigorously validate that the classification performance of our models was significantly above chance and to statistically compare performance across brain regions and model architectures, we performed two distinct statistical analyses: a shuffle control analysis and a standard two-way ANOVA.

First, to validate that the classification performance of our models was significantly above chance and genuinely reflected reward-related information in the neural activity, we performed a shuffle control analysis for each brain region and each of the tuned models. For each analysis, the hold-out test dataset (20% of the data) was used. While keeping the feature vectors and the model's predictions constant, the corresponding trial outcome labels were randomly shuffled 1,000 times. For each iteration, the decoding accuracy was calculated by comparing the model's original predictions against the shuffled labels. This procedure generated a null distribution of accuracy scores, representing the performance expected by chance. To account for multiple comparisons across the 18 conditions (6 brain regions × 3 model architectures), we calculated *p* values based on the aggregated accuracy across the three independent repeats. A Bonferroni’s correction (correction factor, 18) was applied to the uncorrected *p* values.

Second, to examine the hierarchical differences in representation of reward-task–related information, we performed a standard two-way ANOVA with the brain region (six levels) and model architecture (three levels) as factors. For this analysis, individual accuracy scores from the three independent repeats were treated as observations (*N* = 54). This approach was adopted to explicitly incorporate the variability arising from data splitting and model initialization into the statistical evaluation, thereby ensuring that the assessed regional differences are robust to these stochastic factors. The main effect of the brain region was evaluated to determine whether classification performance consistently differed across regions regardless of the model used.

### Interpretation of the final models via SHAP analysis

To interpret the predictions of the final “black box” models, SHAP was employed, a game-theoretic approach that explains the output of any machine learning model (Lundberg and Lee, 2017). Grounded in Shapley values from cooperative game theory, SHAP provides a unified framework for model interpretation with several key advantages. It not only offers global feature importance by averaging the absolute SHAP values across all samples but also provides local, instance-level explanations, revealing how each feature contributes to a specific, individual prediction. This dual capability allows for a comprehensive understanding of model behavior.

For the analysis, SHAP values were computed for each feature and data point in the test set. We visualized the global feature importance using summary plots, which display the distribution of SHAP values for each feature and rank them by their overall impact on the model. To understand the drivers of specific classifications, we used waterfall plots to illustrate how individual feature contributions sum up to push the model's output from the base value to the final prediction for representative single-neuron examples. This approach allowed us to identify the key neural properties that our models learned to associate with rewarded versus nonrewarded trial outcomes.

Since SHAP values are specific to a trained model instance, we selected a representative model for visualization purposes. First, the best-performing model architecture for each brain region was identified based on the mean test accuracy across three independent repetitions. Then, from the three repetitions of this architecture, the specific model instance achieving the highest test accuracy was selected to generate the SHAP summary plots (beeswarm and bar plots) and representative waterfall plots for example neurons. We confirmed that the top-contributing features were largely consistent across the three repetitions.

### Statistical analysis

Standard replication of measurements was performed for this study. The reported findings were reproduced across animals. All quantifications were conducted at the single-neuron level. Sample sizes (the numbers of animals, sessions, and neurons) were estimated according to previous studies (Rios et al., 2019; Sakairi et al., 2025). The KS test was used to extract the test statistic, rather than the *p* value, as a feature for the model. These statistical tests were conducted with MATLAB's Statistics and Machine Learning Toolbox (MathWorks). Blinding and randomization were not performed. Models were tuned via cross-validation on training data, and all reported metrics and interpretability are computed on a held-out test set using the tuned (nonfinalized) model and training-fitted preprocessing.

### Data availability

Data are available upon reasonable request. Data will be made available upon publication in a public repository.

### Code availability

The code/software described in the paper is freely available online at https://github.com/mokamotosan/soma_okamoto_lr_01_public.git. The code is available as Extended Data 1.

**Figure 3.**
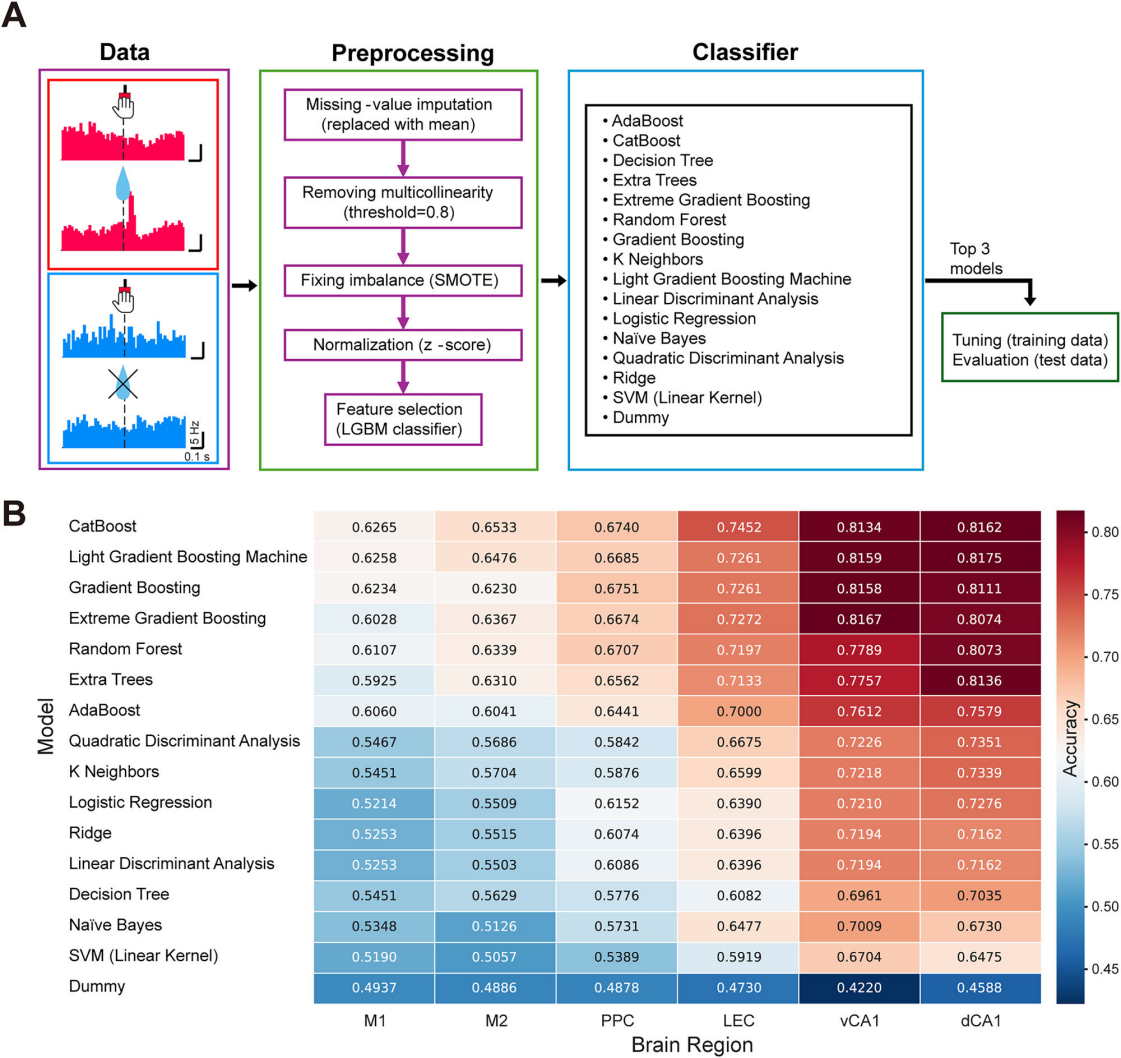
Analysis procedure and model performance. ***A***, Analysis pipeline. A binary classification task was used to determine whether different brain regions retain reward or nonrewarded information relevant to the LR pedal task. Data preparation involved splitting neural spiking data from each neuron into reward trials and nonrewarded trials and averaging the data for each condition. Preprocessing included missing value imputation (replaced with mean), removal of multicollinearity (threshold, 0.8), class imbalance correction (SMOTE), normalization (*z*-score), and feature selection (LightGBM classifier). Subsequently, a variety of classifiers were trained (see Materials and Methods for all the classifiers used), the top three models were tuned, and the final model was evaluated. ***B***, Heatmap of initial model screening results displaying the classification accuracy of 16 machine learning algorithms across six brain regions. Each cell represents the mean accuracy obtained from stratified 10-fold cross-validation. Models (rows) are sorted by their average performance across all regions, while brain regions (columns) are arranged according to the functional hierarchy identified in this study. The color intensity reflects the accuracy level, with warmer colors indicating higher performance. This comprehensive screening was performed using default hyperparameters for all models with a fixed random seed (123) to ensure a fair and reproducible comparison. The heatmap demonstrates a robust hierarchical pattern (dCA1/vCA1 > LEC/PPC > M1/M2) that is consistent across a wide range of algorithmic architectures, from simple linear models to complex ensemble methods. Based on this screening, the top-performing tree–based models (CatBoost, LightGBM, and XGBoost) were selected for subsequent hyperparameter tuning.

**Figure 4.**
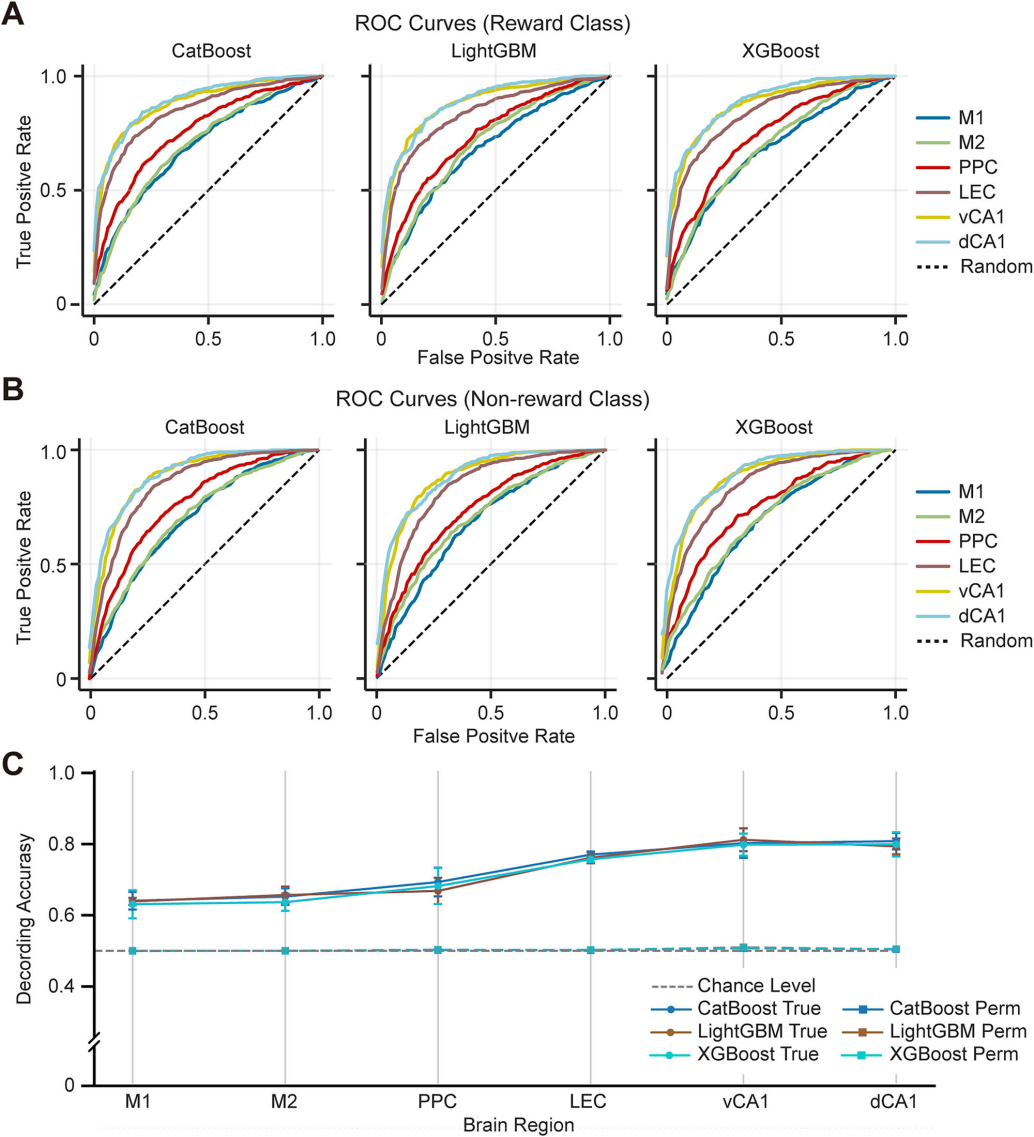
Robust functional hierarchy of reward-related representation revealed by hyperparameter-tuned models. ***A, B***, Receiver operating characteristic (ROC) curves for the classification of rewarded (***A***) and nonrewarded (***B***) trials using the high-performing hyperparameter–tuned models for each brain region. The curves represent the average performance across three independent training/testing repetitions (solid lines). The models displayed are CatBoost, LightGBM, and XGBoost, selected based on the initial screening. A clear performance gradient is visible, with hippocampal regions (dCA1, vCA1) showing the largest area under the curve (AUC), followed by parahippocampal (LEC) and parietal (PPC) regions and finally motor cortices (M1, M2). The diagonal dashed line represents random chance performance (AUC, 0.5). ***C***, Statistical validation of the hierarchical distribution using ANOVA and permutation testing. The line plot compares the actual classification accuracy of the tuned models (true, solid lines/circles) against the baseline accuracy derived from 1,000 permutations of shuffled labels (perm, dashed lines/squares) across the six brain regions. Data points represent the mean accuracy (±SD) calculated separately for each of the three model architectures across three independent repetitions. Classification performance was significantly higher than chance for all region-model pairs (*p* < 0.001; Bonferroni’s corrected; Extended Data Fig. 4-2). Furthermore, a two-way ANOVA (Brain Region * Model) confirmed a highly significant main effect of the brain region (*F*_(5,36)_ = 59.16; *p* < 0.001), statistically substantiating that the observed functional hierarchy (dCA1/vCA1 > LEC/PPC > M2/M1) is a robust property of the neural circuits, independent of the specific machine learning algorithm used (Extended Data Fig. 4-1).

**Figure 5.**
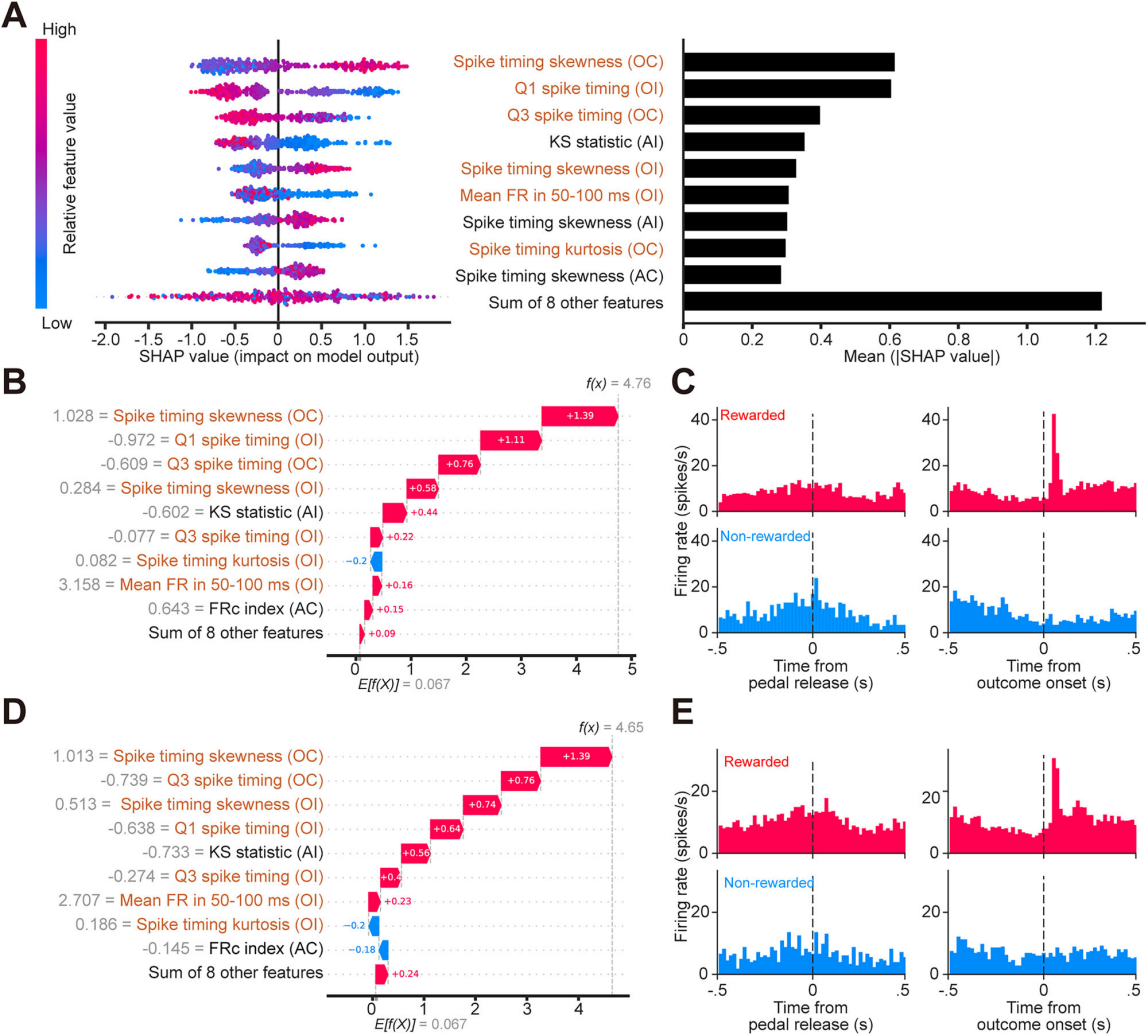
SHAP analysis of the CatBoost model for dCA1. To provide a representative visualization of the feature contributions, the results shown in this figure are derived from the single model instance that achieved the highest classification accuracy among the three independent training/testing repetitions for dCA1 (CatBoost model, repetition 0; see Materials and Methods and Extended Data Fig. 5-1). ***A***, SHAP summary plots. Left, Beeswarm plot showing the impact of each feature on the model output for individual samples (each dot is a sample). The horizontal position indicates the SHAP value, representing the impact on the model's output in log-odds units (margin outputs before the logistic link function); positive values push the prediction toward reward (probability >0.5). Dot color represents the feature value for that sample (red, high; blue, low). Right, Bar plot ranking features by importance (mean absolute SHAP value), with the most influential features at the top. Orange text indicates outcome-related features. Note: the eight least impactful features are collapsed into a single term (“Sum of 8 other features”). ***B, D***, SHAP waterfall plots for single rewarded samples from two example dCA1 neurons (#6654-0 in ***B***, #6684-0 in ***D***). These plots illustrate how the input values of individual features (gray text; corresponding to the dot color in ***A***) contribute to shifting the model's prediction. The length of each bar represents the SHAP value (corresponding to the horizontal position in ***A***), where red bars push the prediction higher (toward reward) and blue bars push it lower, shifting the model's log-odds output *f*(*x*) from the base expected value *E*[*f*(*X*)]. The final model outputs (*f*(*x*) = 4.76 and 4.65, respectively) indicate a high probability of predicting reward, corresponding to probabilities of ∼0.992 for #6654-0 and 0.991 for #6684-0. ***C, E***, Neuronal spiking activity corresponding to the examples in ***B*** and ***D***. Each panel displays PETHs (bin width, 20 ms). Top row, Activity during rewarded trials (red traces) aligned to pedal release (left) and outcome onset (right). Bottom row, Activity during nonrewarded trials (blue traces) aligned to pedal release (left) and outcome onset (right). Dashed lines indicate alignment time points (0 s). Note the sharp increase in spiking activity immediately following outcome onset in rewarded trials (top-right panels) for both example neurons.

**Figure 6.**
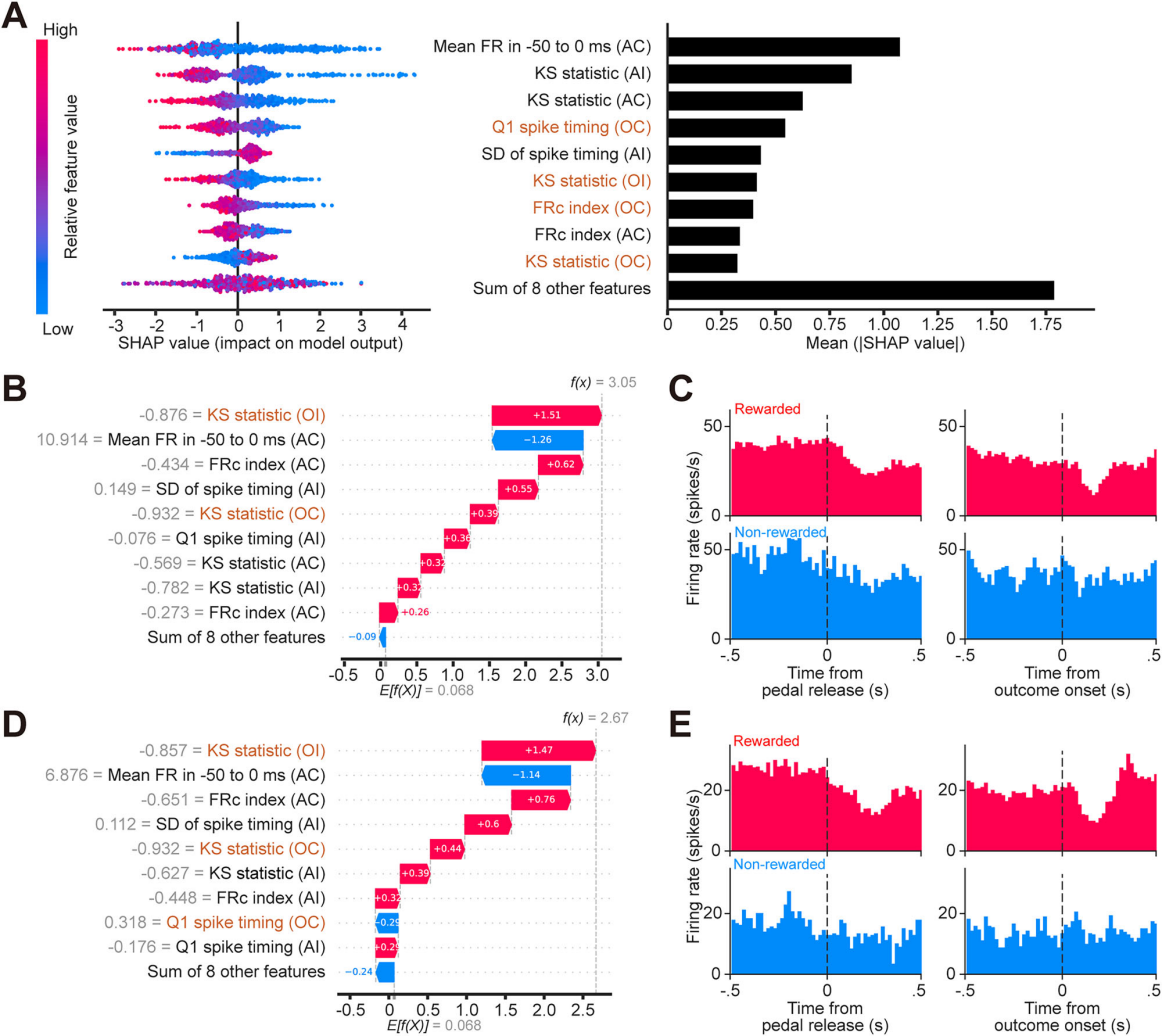
SHAP analysis of the LightGBM model for vCA1. To provide a representative visualization of the feature contributions, the results shown in this figure are derived from the single model instance that achieved the highest classification accuracy among the three independent training/testing repetitions for vCA1 (LightGBM model, repetition 0; see Materials and Methods and Extended Data Fig. 6-1). ***A***, SHAP summary plots. Left, Beeswarm plot showing the impact of each feature on the model output for individual samples (each dot is a sample). The horizontal position indicates the SHAP value, representing the impact on the model's output in log-odds units (margin outputs before the logistic link function); positive values push the prediction toward reward (probability >0.5). Dot color represents the feature value for that sample (red, high; blue, low). Right, Bar plot ranking features by importance (mean absolute SHAP value), with the most influential features at the top. Orange text indicates outcome-related features. Note: the eight least impactful features are collapsed into a single term (“Sum of 8 other features”). ***B, D***, SHAP waterfall plots for single rewarded samples from two example vCA1 neurons (#12260-1 in ***B***, #12424-1 in ***D***). These plots illustrate how the input values of individual features (gray text; corresponding to the dot color in ***A***) contribute (red bars push prediction higher/toward reward; blue bars push lower) to shifting the model's prediction. The length of each bar represents the SHAP value (corresponding to the horizontal position in ***A***), where red bars push the prediction higher (toward reward) and blue bars push it lower, shifting the model's log-odds output *f*(*x*) from the base expected value *E*[*f*(*X*)]. The final model outputs (*f*(*x*) = 3.05 and 2.67, respectively) indicate a high probability of predicting reward, corresponding to probabilities of ∼0.955 for #12260-1 and 0.935 for #12424-1. ***C, E***, The figure legend is the same as in Figure 5, *C* and *E*. Note the decrease in spiking activity immediately following outcome onset in rewarded trials (top-right panels) for both example neurons.

**Figure 7.**
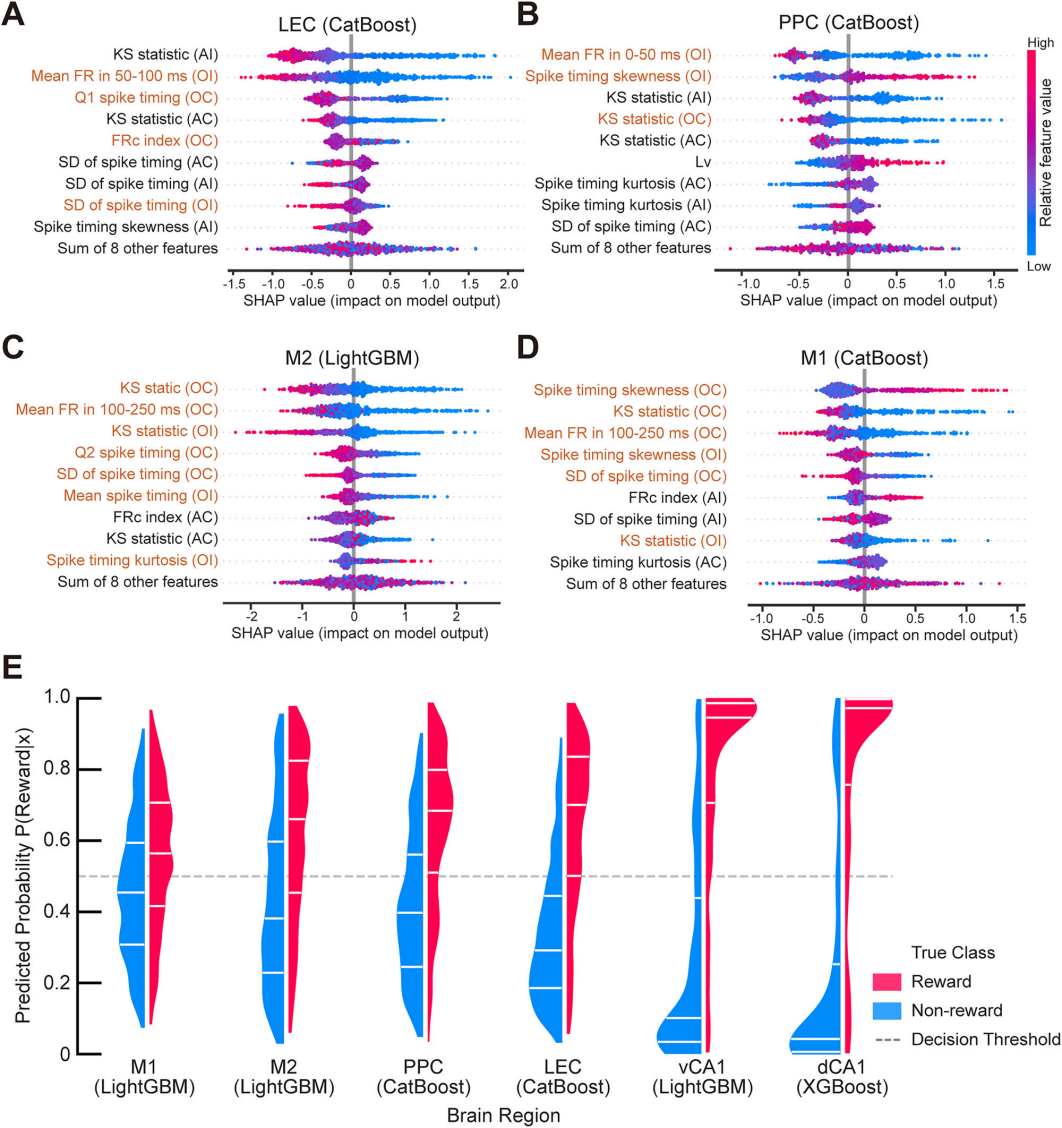
Qualitative transformation of reward-related representation and distinct coding strategies across the cortical–hippocampal hierarchy. ***A–D***, SHAP beeswarm plots revealing the distinct coding strategies in the cortical regions. The plots are derived from the single “best of the best” model instance for each region: LEC (***A***, CatBoost, repetition 0; see also Extended Data Fig. 7-1), PPC (***B***, CatBoost, repetition 0; see also Extended Data Fig. 7-2), M2 (***C***, LightGBM, repetition 0; see also Extended Data Fig. 7-3), and M1 (***D***, CatBoost, repetition 1; see also Extended Data Fig. 7-4). ***E***, Comparison of predicted probability distributions across the six brain regions. Violin plots display the density of the predicted probabilities output by the best-performing models for the test dataset. For consistent comparison, models were selected from a single repetition (repetition 0) based on the highest test accuracy for each region. The distributions are separated by actual trial outcome (rewarded, red; nonrewarded, blue). White horizontal lines within the distributions indicate the quartiles (from bottom to top: first quartile, median, and third quartile). In the hippocampus (dCA1, vCA1), the distributions are sharply polarized near 0 (confident nonreward prediction) and 1 (confident reward prediction), indicating a categorical and high-certainty representation. In M1, the predicted probabilities were concentrated near intermediate values, reflecting a higher degree of uncertainty. M2 displays a broader distribution where the quartiles for rewarded and nonrewarded trials are more distinctly separated compared with M1, despite substantial overlap. LEC and PPC display intermediate profiles, consistent with their transitional roles in the hierarchy.

10.1523/ENEURO.0256-25.2026.d1 Data 1 The complete code, data, and computational environment required to reproduce the findings of this study are available at https://github.com/mokamotosan/soma_okamoto_lr_01_public.git. The repository contains a series of Jupyter Notebooks for analysis (/notebooks) and a Dockerfile to ensure a fully reproducible environment. The analysis is structured with individual notebooks for each of the six brain regions, which generate the results for Figs. 3-7 and Table 1. All analyses were performed within a Docker container based on a Python 3.10 image, utilizing key libraries such as PyCaret for modeling and SHAP for interpretation. To replicate all results, users should first build the Docker environment and then execute the Jupyter Notebooks in the sequence described in the repository's README.md file. Download Data 1, ZIP file.

## Results

### Behavioral task and neural recording

To precisely monitor and measure the timing of behavioral events, we employed a self-initiated left–right choice task (the LR pedal task; Fig. 1*A*,*B*). In this task, head-fixed rats manipulated left and right pedals using the corresponding forelimbs, enabling precise tracking of event timing (action and outcome). Each trial began spontaneously when the rats pressed both pedals down and held them for a self-determined duration. The rats then chose to release either the left or right pedal (action) without any instruction cue in order to obtain saccharin water (reward). The task consisted of two blocks: right pedal rewarded (R, R, R, R…) and left pedal rewarded (L, L, L, L…). The reward pedals alternated block by block without any instruction (Fig. 1*A,B*). Rats typically learned the task within 2 weeks. After learning, neuronal activity was recorded from M1, M2, PPC, dCA1, vCA1, and LEC while rats performed the task (Fig. 1*C*; task performance, 72.0 ± 10.0% in 71 sessions). Analysis windows were aligned to pedal release and outcome onset events (Fig. 1*B*). We analyzed the pre-action and post-outcome epochs (pale yellow areas) but excluded the post-action and pre-outcome periods (gray areas) because they contained movement-related activity (Fig. 1*B*, arrowhead; see Materials and Methods). To visualize temporal dynamics across brain regions, we standardized PETHs for single neurons and averaged across all recorded neurons (Fig. 1*D*). PETHs were aligned to action onset and outcome onset. For each alignment, PETHs were constructed separately for rewarded and nonrewarded trials. Among the recorded brain regions, dCA1 and vCA1 exhibited distinct neuronal dynamics between rewarded and nonrewarded trials, particularly after reward onset: dCA1 showed a strong phasic response following reward delivery, whereas vCA1 exhibited a small peak followed by an inhibitory response. Other regions (M1, PPC, and LEC) displayed only a small peak after reward onset.

To assess whether each of the six brain regions retained task-relevant reward information, we developed and evaluated binary classification models to distinguish between successful and failed trials within each domain by using various neural features (Fig. 2, Extended Data Fig. 2-1).

10.1523/ENEURO.0256-25.2026.f2-1 Figure 2-1 List of the 88 features derived from neuronal activity used for the machine learning classification. These features are grouped into three main categories: fundamental spiking properties, task-related spike-timing properties, and task-related firing-rate properties. Fundamental properties characterize a neuron's intrinsic firing patterns and waveform, using metrics like the coefficient of variation (Cv), local variation (Lv), spike width, and autocorrelogram (ACG) bias. Task-related features quantify neural dynamics around specific events. Spike-timing properties describe the statistical distribution of spike times, including their mean, standard deviation, skewness, kurtosis, and quartiles. Firing-rate properties are calculated from Peri-Event Time Histograms (PETHs) and include mean and peak firing rates in various windows, as well as a firing rate change (FRc) index. These task-dependent features were computed separately for events related to action (A) or outcome (O) and for trials contralateral (C) or ipsilateral (I) to the recording site. Download Figure 2-1, DOCX file.

### Initial model screening to identify top candidates

To efficiently identify the most promising classification algorithms for our data, we first trained a wide array of machine learning models using their default hyperparameters (see Materials and Methods for full list). Their performance was evaluated using 10-fold cross-validation across all brain regions. The goal of this initial step was to select a small subset of models that performed well generally, allowing us to focus subsequent tuning efforts efficiently (Fig. 3*A*). To visualize the comprehensive performance landscape, we compiled the accuracy of all 16 classifiers across all six regions into a heatmap (Fig. 3*B*). This analysis revealed two key insights.

First, three classifiers—CatBoost, LightGBM, and XGBoost—consistently exhibited superior performance compared with other algorithms across all regions (Fig. 3*B*). Based on this consistent superiority, these three models were selected for further optimization and analysis. Second, the heatmap demonstrates that the performance hierarchy (vCA1/dCA1 > LEC/PPC > M1/M2) is largely robust to the choice of classification algorithm. For instance, even the lower-performing models in dCA1 (e.g., SVM; accuracy = 0.6475) were comparable to or outperformed the very best models in M1 and M2 (e.g., M2 CatBoost = 0.6533; M1 CatBoost = 0.6265). In fact, only two models in M2 (CatBoost and LightGBM) marginally exceeded the performance of the lowest-ranked classifier in dCA1 (excluding the dummy classifier). This consistency confirms that the observed functional hierarchy reflects intrinsic differences in the information content of these brain regions rather than being an artifact of specific algorithm selection.

### Hyperparameter tuning and assessing hierarchy robustness across top models

Having identified CatBoost, LightGBM, and XGBoost as top candidates, hyperparameter tuning was performed for each to optimize their performance based on accuracy (see Materials and Methods). The intent here was to assess whether the relative performance differences observed across the six brain regions were robust and not merely an artifact of a single algorithm. Table 1 summarizes the generalization performance (Accuracy and AUC) of these three “tuned” models for each brain region, evaluated on the held-out test set. Crucially, all three tuned models consistently showed the same performance hierarchy: the highest accuracy and AUC were achieved for vCA1 and dCA1, followed by LEC and PPC, with M1 and M2 performing lowest. This consistent ranking across the three independently tuned, high-performing models strongly suggests that the observed hierarchy reflects genuine differences in representation of reward-related information across the brain regions rather than algorithm-specific biases. The ROC curves for the tuned models further illustrate this hierarchy (Fig. 4*A*,*B*), with curves for vCA1 and dCA1 clearly positioned above those for other regions. Note that the ROC curves for all three models were positioned notably above the diagonal line, which represents the performance of a random classifier. This positioning indicates that all three models demonstrated strong discriminative ability, effectively distinguishing between the classes of interest.

10.1523/ENEURO.0256-25.2026.f4-1 Figure 4-1 Summary table of the two-way analysis of variance (ANOVA) performed to assess the effects of Brain Region and Model Architecture on classification accuracy. The analysis treated individual test accuracy scores from three independent training/testing repetitions as observations (N = 54). Factors: Brain Region (6 levels: M1, M2, PPC, LEC, vCA1, dCA1) and Model Architecture (3 levels: CatBoost, LightGBM, XGBoost). Results: A highly significant main effect of Brain Region was observed (*F*_(5,36)_ = 59.16, *p* < 0.0001), confirming the presence of a statistically distinct performance gradient across regions. In contrast, neither the main effect of Model Architecture (*F*_(2,36)_ = 0.57, *p* = 0.57) nor the interaction between Region and Architecture (*F*_(10,36)_ = 0.19, *p* = 0.99) was significant. These results statistically demonstrate that the observed functional hierarchy (Hippocampus > Parahippocampal/Parietal > Motor) is a robust biological property that persists regardless of the specific machine learning algorithm used**.** Download Figure 4-1, DOCX file.

10.1523/ENEURO.0256-25.2026.f4-2 Figure 4-2 The table summarizes the statistical validation of the hyperparameter-tuned models, comparing their actual classification accuracy (“True accuracy”) against chance-level performance distributions derived from shuffle control analyses (“Permutation accuracy”). For each of the 18 conditions (comprising 6 brain regions and 3 model architectures), trial outcome labels were randomly shuffled 1,000 times to generate a null distribution. “True accuracy” denotes the model's performance on the original test set, while “Permutation accuracy” reports the mean and standard deviation (SD) of accuracy scores from the shuffled datasets. Statistical significance (p-value) was determined with Bonferroni correction for multiple comparisons (correction factor = 18). A p-value of 0.0180 represents the resolution limit of this permutation test (1/1,000 iterations × 18 comparisons), indicating that the true accuracy surpassed the accuracy of all 1,000 permuted samples (uncorrected *p* < 0.001) in every instance. These results confirm that classification performance was significantly above chance across all regions and models. Download Figure 4-2, DOCX file.

### Robustness of functional hierarchy confirmed by ANOVA and shuffled control

To verify the robustness of the observed functional hierarchy and to rigorously confirm that the models were decoding genuine reward-related signals rather than spurious correlations, we performed comprehensive statistical validations. First, to statistically substantiate the hierarchical differences observed across regions, we performed a two-way ANOVA with brain region and model architecture as factors (see Materials and Methods). The analysis revealed a highly significant main effect of the brain region (*F*_(5,36)_ = 59.16; *p* < 0.001), statistically confirming the existence of a performance gradient. Crucially, neither the main effect of model architecture (*F*_(2,36)_ = 0.57; *p* = 0.57) nor the interaction between region and model (*F*_(10,36)_ = 0.19; *p* = 0.99) was significant (Extended Data Fig. 4-1). This lack of significant interaction provides compelling statistical evidence that the observed functional hierarchy (hippocampus > association > motor) is a robust biological property rather than being an artifact of specific algorithm biases. Furthermore, we confirmed that all models captured meaningful information. A shuffle control analysis with Bonferroni’s correction demonstrated that the classification accuracy for every model–region pair was significantly higher than the chance level (*p* < 0.05; see Materials and Methods, Statistical verification of model performance: shuffle control analysis and ANOVA; Fig. 4*C*, Extended Data Fig. 4-2). This result provides strong evidence that the observed decoding performance and the resulting functional hierarchy are driven by true neural representations of trial outcomes.

### Interpretation of the final models

To understand which neuronal features contributed to the high classification performance, we utilized SHAP analysis. As detailed in the Materials and Methods (see Interpretation of the final models via SHAP analysis), for each brain region, the “final model” architecture was determined based on the highest mean accuracy across three independent training/testing repetitions (Table 1). Specifically, the best-performing models were identified as follows: CatBoost for dCA1, LightGBM for vCA1, CatBoost for LEC, CatBoost for PPC, LightGBM for M2, and CatBoost for M1. To robustly identify the most critical features, we selected “top-contribution features” as those that consistently ranked within the top nine mean absolute SHAP values across all three repetitions for the selected model architecture.

For visualization purposes (SHAP beeswarm plots, bar plots of mean absolute SHAP values, and waterfall plots for representative neurons), we presented the results from the single “best of the best” model instance—the specific repetition (out of three) that achieved the highest test accuracy for that region. These specific models were CatBoost (repetition 0) for dCA1, LightGBM (repetition 0) for vCA1, CatBoost (repetition 0) for LEC, CatBoost (repetition 0) for PPC, LightGBM (repetition 0) for M2, and CatBoost (repetition 1) for M1.

### dCA1 model interpretation

The SHAP analysis revealed that features related to the “temporal distribution of spikes following reward delivery” were most influential. Consistent across all three independent repetitions, features such as spike timing skewness (both OC and OI) and spike timing kurtosis (OC) consistently ranked among the top contributors (Extended Data Fig. 5-1). The model also utilized rate-based information, specifically the transient increase in firing rate shortly after outcome onset (mean FR in 50–100 ms for OI). Figure 5*A* shows the contributions of those features to the prediction of the model instance achieving highest test accuracy across three repetitions. In addition to these stable features, the precise timing of spikes relative to the reward onset played a critical role in the best-performing model instance shown in Figure 5. Specifically, the first and third quartiles of the spike timing distribution (Q1 spike timing and Q3 spike timing) were identified as top-ranking predictors (second and third highest importance, respectively, in Fig. 5*A*). While these specific quartile features showed some variability in ranking across repetitions—falling just outside the top nine in one instance (repetition 2) despite being highly ranked in others (repetition 0 and 1)—their exceptionally high contribution in the best-performing model further underscores the importance of temporal structure in hippocampal coding. These SHAP results indicate that the model heavily relies on the pattern of spiking immediately after reward. This aligns well with neurophysiological observations. Example dCA1 neurons, for which the model predicted a high probability of reward (Fig. 5*B,D*), showed that these same temporal features made large contributions to the prediction. Correspondingly, the PETHs for these neurons exhibited a sharp, transient increase in the firing rate immediately following reward delivery (Fig. 5*C*,*E*, top-right panels), matching the pattern seen in the grand-averaged PETH (Fig. 1*D*, dCA1). Thus, the model effectively learned that this early, concentrated burst of spikes, captured by positive skewness and low quartile values, is characteristic of rewarded trials in dCA1.

10.1523/ENEURO.0256-25.2026.f5-1 Figure 5-1 This table summarizes the classification performance and the top-ranking features for the best model architecture (CatBoost) in the dorsal CA1 (dCA1) region across three independent training/testing repetitions (Repeat 0, 1, and 2). The best model architecture was determined based on the highest mean accuracy across repetitions (see Materials and Methods). For each repetition, the table lists the performance metrics (Accuracy and AUC) on the held-out test set, with the maximum values across repetitions indicated by asterisks (*). The top 9 features with the highest mean absolute SHAP values are listed in descending order of importance. Features that consistently ranked within the top 9 across all three repetitions are highlighted in bold text. This consistency underscores the robust contribution of specific spike timing distribution features (e.g., skewness, quartiles) to the model's predictions in dCA1, confirming that these coding strategies are stable biological properties rather than artifacts of specific data splits or model initializations. Download Figure 5-1, DOCX file.

### vCA1 model interpretation

Here, the SHAP analysis indicated a more complex picture, with influential features related to both action timing and outcome processing. Consistent across all three independent repetitions, action-related features such as KS statistic for both ipsilateral and contralateral action trials [KS statistic (AI) and KS statistic (AC)] consistently ranked among the top three contributors. Notably, the KS statistic for outcome-related trials [KS statistic (OI)] also contributed significantly across repetitions, reflecting the importance of spike timing distribution uniformity throughout the 500 ms window before and after reward delivery. In the best-performing model instance shown in Figure 6, *A* and *B*, additional outcome-related features played a prominent role. Specifically, the first quartile of post-reward spike timing [Q1 spike timing (OC)] ranked highly, where lower values pushed predictions toward reward, similar to the pattern observed in dCA1. Although this feature was not ranked in the top nine in repetition 1, it was a top contributor in both repetition 0 (best model) and repetition 2. Furthermore, the FRc index after reward (OC) also emerged as a top predictor in the best model (Extended Data Fig. 6-1). Examining example vCA1 neurons highlights the role of outcome-related features, particularly the spike timing distribution indices like KS statistic and Q1 spike timing (Fig. 6*B*,*D*). The corresponding PETHs often showed a clear suppression of firing activity following reward delivery (Fig. 6*C*,*E*, top-right panels), contrasting with the excitatory response in dCA1.

10.1523/ENEURO.0256-25.2026.f6-1 Figure 6-1 This table summarizes the classification performance and the top-ranking features for the best model architecture (LightGBM) in the ventral CA1 (vCA1) region across three independent training/testing repetitions (Repeat 0, 1, and 2). The best model architecture was determined based on the highest mean accuracy across repetitions (see Materials and Methods). For each repetition, the table lists the performance metrics (Accuracy and AUC) on the held-out test set, with the maximum values across repetitions indicated by asterisks (*). The top 9 features with the highest mean absolute SHAP values are listed in descending order of importance. Features that consistently ranked within the top 9 across all three repetitions are highlighted in bold text, indicating the robust contribution of specific action- and outcome-related statistics (e.g., KS statistics). Notably, although not present in all three repetitions, the first quartile of post-reward spike timing (Q1 spike timing (OC)) ranked highly in two out of three repetitions (Repeat 0 and 2), including the best-performing instance (Repeat 0), further supporting the relevance of temporal coding features in this region. Download Figure 6-1, DOCX file.

### Distinct coding strategies in cortical regions

To address whether the observed performance hierarchy reflects qualitative differences in neural coding strategies, we extended our SHAP analysis to the cortical regions (LEC, PPC, M1, and M2; Fig. 7*A*–*D*). We applied the same selection criteria used for the hippocampal regions, choosing the best-performing model instance for each region to visualize feature importance.

10.1523/ENEURO.0256-25.2026.f7-1 Figure 7-1 This table summarizes the classification performance and the top-ranking features for the best model architecture (CatBoost) in the lateral entorhinal cortex (LEC) across three independent training/testing repetitions (Repeat 0, 1, and 2). The best model architecture was determined based on the highest mean accuracy across repetitions (see Materials and Methods). For each repetition, the table lists the performance metrics (Accuracy and AUC) on the held-out test set, with the maximum values across repetitions indicated by asterisks (*). The top 9 features with the highest mean absolute SHAP values are listed in descending order of importance. Features that consistently ranked within the top 9 across all three repetitions are highlighted in bold text. Notably, the FRc index (outcome-related firing rate change) consistently appeared as a top feature, reflecting the dominance of rate-based coding (suppression) in this region. Additionally, temporal features such as Q1 spike timing and KS statistic ranked within the top 9 in two out of three repetitions (including the best-performing Repeat 0), suggesting that while rate coding is primary, the LEC also exhibits emerging temporal coding properties, consistent with its role as a transitional gateway to the hippocampus. Download Figure 7-1, DOCX file.

10.1523/ENEURO.0256-25.2026.f7-2 Figure 7-2 This table summarizes the classification performance and the top-ranking features for the best model architecture (CatBoost) in the posterior parietal cortex (PPC) across three independent training/testing repetitions (Repeat 0, 1, and 2). The best model architecture was determined based on the highest mean accuracy across repetitions (see Materials and Methods). For each repetition, the table lists the performance metrics (Accuracy and AUC) on the held-out test set, with the maximum values across repetitions indicated by asterisks (*). The top features with the highest mean absolute SHAP values are listed in descending order of importance. Features that consistently ranked within the top list across all three repetitions are highlighted in bold text. Common features consistently identified include Mean Firing Rate in 0–50 ms, spike timing skewness (OI), KS statistic (OC), and Lv. Notably, the consistent contribution of Lv, a metric of intrinsic firing regularity, implies that the model relies not only on task-evoked responses but also on the intrinsic properties of neurons, suggesting that specific cell types with distinct firing characteristics play a significant role in encoding reward information in the PPC. Download Figure 7-2, DOCX file.

10.1523/ENEURO.0256-25.2026.f7-3 Figure 7-3 This table summarizes the classification performance and the top-ranking features for the best model architecture (LightGBM) in the secondary motor cortex (M2) across three independent training/testing repetitions (Repeat 0, 1, and 2). The best model architecture was determined based on the highest mean accuracy across repetitions (see Materials and Methods). For each repetition, the table lists the performance metrics (Accuracy and AUC) on the held-out test set, with the maximum values across repetitions indicated by asterisks (*). The top 9 features with the highest mean absolute SHAP values are listed in descending order of importance. Features that consistently ranked within the top 9 across all three repetitions are highlighted in bold text. Common features consistently identified include KS statistic (OC,OI) and SD of spike timing (OC). The robust contribution of high-order statistical features like the KS statistic (quantifying distribution uniformity) and SD of spike timing, rather than simple firing rate magnitude, reflects the “subtle and distributed” coding strategy characteristic of M2. Note that the classification accuracy in M2 is markedly lower compared to the hippocampal (dCA1, vCA1) and parahippocampal (LEC) regions, supporting the functional hierarchy described in the main text. Download Figure 7-3, DOCX file.

10.1523/ENEURO.0256-25.2026.f7-4 Figure 7-4 This table summarizes the classification performance and the top-ranking features for the best model architecture (CatBoost) in the primary motor cortex (M1) across three independent training/testing repetitions (Repeat 0, 1, and 2). The best model architecture was determined based on the highest mean accuracy across repetitions (see Materials and Methods). For each repetition, the table lists the performance metrics (Accuracy and AUC) on the held-out test set, with the maximum values across repetitions indicated by asterisks (*). The top 9 features with the highest mean absolute SHAP values are listed in descending order of importance. Features that consistently ranked within the top 9 across all three repetitions are highlighted in bold text. Common features consistently identified include spike timing skewness (OC and OI) and KS statistic (OC and OI). Similar to M2, the classification accuracy in M1 is substantially lower than in the hippocampal and parahippocampal regions, consistent with its position at the lower end of the functional hierarchy. Download Figure 7-4, DOCX file.

In the LEC, the FRc index for contralateral outcome emerged as a key feature across repetitions (Fig. 7*A*; Extended Data Fig. 7-1). The SHAP summary plot indicated that lower values of this feature were associated with the rewarded class. This is consistent with the grand-averaged PETH for LEC (Fig. 1*D*), which showed a suppression of firing activity (i.e., decreased firing rate relative to baseline) following reward delivery. Thus, the model successfully learned to utilize this reward-induced suppression—manifesting as negative FRc index values—as a discriminative signal for trial outcome. It is noteworthy that in two out of the three independent repetitions (including the best-performing model shown in Fig. 7), timing-related features such as Q1 spike timing and KS statistic also ranked within the top-contributing features. This suggests that while rate modulation is the dominant mode of information representation in the LEC, there are emerging signs of temporal coding. This hybrid profile supports the view that the LEC may serve as a transitional stage where a transformation from rate-based to sparse, temporal coding begins to occur at the entry to the hippocampus.

In the PPC, across the three independent repetitions, four features consistently ranked among the top predictors: mean FR in 0–50 ms, spike timing skewness (OI), KS statistic (OC), and Lv (Fig. 7*B*; Extended Data Fig. 7-2). Notably, three of these four features (mean FR in 0–50 ms, skewness, and KS statistic) capture the precise timing or transient distribution of spikes relative to the reward onset. This indicates that the PPC utilizes temporal information related to the immediate processing of reward outcomes. It is noteworthy that Lv, a metric of intrinsic firing regularity often used to distinguish cell types (e.g., bursty vs regular spiking neurons), emerged as a consistent top predictor, suggesting that the model not only utilizes task-evoked responses but also relies on intrinsic firing properties to weight the contributions of specific cell types.

In the M2, the coding strategy appeared to be the most subtle and distributed among the analyzed regions (Fig. 7*C*; Extended Data Fig. 7-3). In M2, the top-ranking features consistently included the KS statistic (for both OI and OC) and the SD of spike timing (OC). The KS statistic quantifies the deviation of spike timing from a uniform distribution, effectively capturing temporal nonuniformity or bias in spiking activity within the analysis window.

Similarly, in M1, the model relied on higher-order statistics such as spike timing skewness and KS statistic (for both OI and OC; Fig. 7*D*; Extended Data Fig. 7-4). Unlike M2, the M1 PETH shows a slight but observable transient increase in the firing rate immediately after reward delivery. Consistent with this, the SHAP analysis indicated that higher skewness in contralateral trials (reflecting a distribution biased toward earlier spikes) contributed to predicting the rewarded class.

#### Distributions of predicted probabilities across regions

To characterize the nature of reward-related representation beyond simple classification accuracy, we examined the distributions of the predicted probabilities output by the best-performing models for the test data (Fig. 7*E*). The distributions revealed a striking contrast between the hippocampus and the neocortex. In the hippocampal regions (dCA1 and vCA1), the probability distributions for rewarded and nonrewarded trials were clearly separated, with most predictions concentrated near the extremes of 0 (nonrewarded) and 1 (rewarded). This indicates that the models distinguished the trial outcomes with high confidence and a clear decision margin.

In contrast, the neocortical regions exhibited more graded and overlapping distributions. In M1, the predicted probabilities were concentrated near intermediate values, reflecting a higher degree of uncertainty in the classification. M2 also showed substantial overlap between classes similar to M1 but displayed a broader distribution with a distinct skew. Specifically, a comparison of the quartiles (indicated by white lines) reveals that the distribution for nonrewarded trials extended more toward the lower range (first quartile), whereas rewarded trials shifted toward the higher range (third quartile). This suggests that although overall classification performance remains comparable between the two regions, M2 exhibits a slightly more differentiated probability structure compared with the highly centralized uncertainty observed in M1. The LEC and PPC displayed intermediate profiles, illustrating a progressive transition from the motor cortices to the hippocampus. In the progression from PPC to LEC, a systematic shift in the distribution statistics was observed. For nonrewarded trials, the median and first quartile shifted progressively toward the lower range, with the distribution peaks moving accordingly and the tails becoming lighter. Conversely, for rewarded trials, the median and third quartile shifted toward the higher range, with peaks moving upward and tails becoming lighter. Consequently, while the PPC still exhibited broadly overlapping distributions similar to the motor areas, the LEC demonstrated a more distinct separation, effectively bridging the gap between the graded uncertainty of the motor cortices and the categorical certainty of the hippocampus. These results indicate that while classification is possible in all regions, the “certainty” and categorical nature of the information representation vary distinctively across the hierarchy.

## Discussion

In this study, the neural representation of reward-related information was quantitatively compared across six key brain regions—ranging from motor and parietal cortices to the parahippocampal and hippocampal structures—using a unified machine learning framework. A comprehensive analysis of single-neuron activity yielded three main findings. First, while all examined regions contained information sufficient to classify rewarded from nonrewarded trials significantly above chance, a clear functional hierarchy in predictive performance was observed: the hippocampus (dCA1 and vCA1) exhibited the highest accuracy, followed by the parahippocampal region (LEC) and parietal cortex (PPC), with the motor cortices (M1 and M2) showing the lowest performance (Fig. 4). Second, this hierarchical order was robust across a wide variety of machine learning algorithms (Fig. 4*C*), suggesting it reflects an intrinsic property of the neural circuits rather than a model-specific artifact. Third, and most crucially, SHAP-based model interpretation revealed that this performance gradient corresponds to distinct, region-specific coding strategies (Figs. 5–7). A qualitative transformation of information processing was found: from subtle, distributed, and high-order statistical representations in the motor cortices to heterogeneous and transitional representations in the PPC and LEC and finally culminating in precise, temporal, and polarity-specific (excitatory vs suppressive) representations in the dorsal and ventral hippocampus (Fig. 7)

### Robustness of functional hierarchy across classification algorithms

A critical question in decoding analysis is whether the observed performance differences reflect true biological distinctions or are merely artifacts of specific model architectures. Our comprehensive screening of 15 diverse algorithms (Fig. 3*B*) addresses these concerns by demonstrating that the functional hierarchy (dCA1/vCA1 > LEC/PPC > M1/M2) is remarkably robust.

First, the relative ranking of brain regions remained invariant across all 15 models, regardless of their algorithmic complexity (linear vs nonlinear). Notably, even the lower-performing models in dCA1 (e.g., SVM) achieved accuracy comparable to or exceeding the very best-performing models in the motor cortices (e.g., M2 CatBoost). This indicates that the information gap between these regions is substantial enough to overcome algorithmic performance differences.

Second, we ruled out artifacts arising from the binary classification framework by comparing classifiers with fundamentally different mathematical assumptions. We reasoned that if the observed hierarchy were merely an artifact of the binary task structure favoring specific algorithms (e.g., SVM), then classifiers based on different principles, such as LDA (typically used for multiclass problems), should exhibit divergent regional rankings. However, contrary to this prediction, both SVM (binary-optimized) and LDA (multiclass-optimized) exhibited the same regional trends, confirming that our findings are not driven by task structure.

Finally, while the hierarchical trend was universal, tree-based ensemble methods (CatBoost, LightGBM, XGBoost) consistently achieved the highest absolute accuracy across all regions. This specific performance advantage likely stems from their inherent ability to capture complex, nonlinear interactions among neural features that linear models miss, allowing for a more precise readout of the underlying neural code.

### Distinct coding strategies across regions

The SHAP analysis went beyond simple performance metrics to uncover a diversity of coding strategies that parallels the performance hierarchy.

### Hippocampus: the apex of temporal and suppressive coding

The hippocampus stood at the top of the functional hierarchy, achieving the highest classification accuracy. However, vCA1 and dCA1 achieved this through strikingly different yet complementary strategies. SHAP analysis revealed that the dCA1 model primarily relied on features describing the temporal distribution of spikes immediately following reward delivery. Specifically, positive skewness (early clustering of spikes) and low quartile values (indicating spike concentration near reward onset) were highly predictive of rewarded trials (Fig. 5). This emphasis on early, temporally concentrated spiking aligns with PETH observations (Figs. 1*D*, 5*C*,*E*), which exhibited increased firing immediately after reward. This precise temporal structuring allows dCA1 to represent reward events with high fidelity and low latency.

In contrast, the vCA1 model utilized a multifaceted strategy. Robustness analysis consistently identified the KS statistic as a dominant feature, underscoring the fundamental role of spike timing modulation. This temporal code was complemented by the FRc index, whose negative contribution in the best-performing model aligned with the transient firing suppression observed in PETHs. Specifically, SHAP analysis showed that lower values of the FRc index (OC) were predictive of rewarded trials (Fig. 5*A*), mirroring the transient firing suppression observed immediately after reward onset in PETHs (Fig. 6*C*,*E*). Thus, vCA1 signals reward-related information by integrating distinct spike timing distributions with a reduction in firing activity.

It is noteworthy that the dorsal and ventral hippocampi distinctly represent environmental information and differentially modulate downstream networks with their unique temporal dynamics (Royer et al., 2010; Sosa et al., 2020). The dorsal hippocampus is well known for encoding reward-related information (Singer and Frank, 2009; Gauthier and Tank, 2018; Kaufman et al., 2020; Robinson et al., 2020; Sato et al., 2020; Soma et al., 2023; Sakairi et al., 2025). It is suggested that the intrinsic activity may guide goal-directed navigation and behavior (Sosa and Giocomo, 2021).

The ventral hippocampus, on the other hand, is implicated in diverse functions ranging from social memory (Okuyama et al., 2016; Rao et al., 2019) and fear memory (Jimenez et al., 2018, 2020; Kim and Cho, 2020) to reward-related behaviors (Ruediger et al., 2012; Riaz et al., 2017; Kosugi et al., 2021; Chen et al., 2023) and sensory/sensorimotor gating (Swerdlow et al., 2004, 2012; Okamoto et al., 2012). Manipulations of vCA1 activity significantly impact these behaviors (Riaz et al., 2017; Yoshida et al., 2019). Furthermore, the neuronal response in vCA1 to rewards are known to be circuit-specific (Kosugi et al., 2021; Chen et al., 2023), highlighting the complexity of its role. Notably, inhibitory mechanisms within the ventral hippocampus are considered crucial for the functions like sensory/sensorimotor gating (Okamoto et al., 2012). Intriguingly, in the current study, vCA1 neurons showed suppressive responses after reward delivery. While its function remains unclear, this suppression may relate to gating mechanisms or context-dependent signaling. Understanding how this reward-related suppression affects behavior will be important for future research.

### LEC: the integrative gateway

The LEC, serving as a primary interface between the neocortex and the hippocampus, displayed a “transitional” coding strategy. The dominant feature for reward prediction was the FRc index (specifically for contralateral outcomes), where firing suppression contributed to reward identification. However, unlike the motor areas, temporal features such as Q1 and KS statistics also began to emerge as important predictors. This hybrid profile positions the LEC as an “integrative gateway,” potentially transforming the distributed, rate-based signals from upstream cortical areas into a format more suitable for the temporal processing mechanisms of the hippocampus (Doan et al., 2019; Nilssen et al., 2019).

### PPC: heterogeneous population coding

The PPC exhibited intermediate performance and a more complex coding profile. While the population-averaged PETH showed a general excitatory response to reward, the SHAP analysis also revealed complex relationships characteristic of a functionally heterogeneous population. For instance, while specific temporal features like skewness pushed predictions toward reward (Fig. 7*B*), higher values of mean FR (in the 0–50 ms window) were often associated with the nonrewarded class in the summary plot. This stands in contrast to the population-averaged PETH, which shows a clear excitatory response to reward (Fig. 1*D*). This discrepancy likely reflects the functional heterogeneity of PPC neurons; the machine learning model may have identified specific subpopulations—likely those exhibiting reward-induced suppression or weaker responses compared with error trials—as robust predictors for the error class. This hypothesis is further supported by the high contribution of Lv (Fig. 7*B*). Lv is a metric of intrinsic firing regularity often used to distinguish cell types (e.g., bursty vs regular spiking neurons; Shinomoto et al., 2009). The fact that Lv emerged as a consistent top predictor suggests that the model not only utilizes task-evoked responses but also relies on intrinsic firing properties to weight the contributions of specific cell types.

### M2: subtle and distributed coding via high-order statistics

The M2 presented a unique profile. Despite showing almost no discernible difference in mean FR between rewarded and nonrewarded trials during the post-outcome period (Fig. 1*D*), the machine learning models successfully predicted reward outcomes above chance. SHAP analysis indicated a reliance on high-order statistical features of the spike trains, such as the KS statistic and SD of spike timing (Fig. 7*C*), rather than simple rate changes. This suggests a “subtle and distributed” coding strategy, where information is embedded in the fine temporal structure or regularity of spiking activity rather than robust rate modulation. This reliance on such nuanced, higher-order statistics likely explains why M2 occupied the lower tier in the classification performance hierarchy.

### M1: direction-dependent and embodied representation

The M1 exhibited the lowest predictive power among all regions, despite showing a transient increase in the firing rate following reward delivery (Fig. 1*D*). SHAP analysis revealed a complex, “direction-dependent” coding strategy: for ipsilateral trials, higher skewness pushed predictions toward the nonrewarded class, and higher KS statistical values (indicating greater nonuniformity) were generally associated with error trials. This complexity likely arises because M1 activity reflects not only abstract reward information but also lateralized motor preparations or subtle movements associated with consuming the reward. The fact that the model had to leverage such intricate and direction-dependent dependencies explains why M1 showed the lowest performance.

### A hierarchical function/structure across the cortical and hippocampal regions

The observed performance hierarchy aligns well with the putative roles of these areas in the cognitive components of our task—outcome monitoring, decision-making, and motor execution. The hippocampus exhibited the highest predictive performance, consistent with its central role in encoding ongoing events into working and episodic memory (Yonelinas, 2013; Eichenbaum, 2017). Additionally, the short latency of reward-related responses (<100 ms) observed in dCA1 supports its involvement in real-time event representation (Sakairi et al., 2025). The strong performance of the LEC, a major hippocampal–neocortical interface (Witter et al., 2017; Ohara et al., 2021), further supports this view. Notably, the LEC receives direct inputs from the PPC and motor cortices (Burwell and Amaral, 1998; Olsen et al., 2017), and both dCA1 and LEC receive direct dopaminergic input from VTA/SNc (Edelmann and Lessmann, 2018; Lee et al., 2021; Tsetsenis et al., 2021), positioning them as potential dopamine-modulated hubs linking past actions and its outcomes to future decisions. The PPC's intermediate performance likely reflects, despite relatively sparse dopaminergic innervation (Berger et al., 1991), its established role in decision-making (Harvey et al., 2012; Raposo et al., 2014; Goard et al., 2016) and its inputs from dopamine-rich areas like the orbitofrontal cortex (Whitlock, 2017). Finally, motor cortices, which also project to the VTA/SNc (Watabe-Uchida et al., 2012), showed the lowest yet still above-chance performance. This aligns with prior findings of reward-related activity (Ramkumar et al., 2016; Ramakrishnan et al., 2017) and suggests involvement beyond pure motor execution, potentially in adjusting motor output based on reward context (Derosiere et al., 2025). The consistently better performance of M2 over M1 in our models may reflect M2's role in representing more abstract movement features compared with M1's encoding of specific commands (Soma et al., 2017), revealing a functional distinction even within the motor system for this task.

### Strengths, limitations, and future directions

A key contribution of this study is the direct, quantitative comparison of representation of reward-related information across six distinct brain regions. By employing identical behavioral, recording, and machine learning methodologies, we provide an unbiased assessment of the relative robustness of reward-related information coding. This approach not only confirmed the widespread distribution of such information but, crucially, revealed a clear performance hierarchy. This functional hierarchy is particularly intriguing when considered in light of recent reports demonstrating a correlation between electrophysiologically derived functional hierarchy and anatomical hierarchy (Siegle et al., 2021; Song et al., 2024). These findings are based on hierarchy scores obtained from large-scale corticothalamic tracing experiments (Harris et al., 2019), shedding light on the hierarchy in visual function and its potential for temporal representation. Unfortunately, only M1 and M2 scores have been reported in these large-scale corticothalamic tracing experiments, which prevent a comprehensive comparison with all the regions in this study. However, at least for these two regions, the functional hierarchy and the anatomically derived hierarchy scores were consistent (M1 < M2; e.g., hierarchy score; M1 = −0.05; M2 = 0.18; Harris et al., 2019; AUC in Table 1). In the future, by comparing the anatomical hierarchy scores of the hippocampal formation and parietal association cortex with our functional hierarchy, we aim to integrate insights from both machine learning results of physiological data and hierarchical score of anatomical data.

Furthermore, the use of interpretable machine learning (SHAP analysis) provided a key methodological strength, allowing us to uncover the distinct coding strategies underlying reward-related information in the dorsal and ventral hippocampus. The analysis revealed that dCA1 relies heavily on the precise temporal distribution of post-reward spikes, whereas vCA1 integrates a broader set of spike timing and firing rate features (Figs. 5, 6). This level of insight was enabled by our comprehensive approach to feature selection. As outlined in the Materials and Methods, task-independent intrinsic properties (e.g., spike width, Cv) were intentionally included to test the hypothesis that a neuron's cell type, in combination with its task-dependent responses, could be predictive of its role. The SHAP analysis, in turn, revealed that the most influential predictors in the hippocampus were predominantly these dynamic, task-dependent features. While this does not exclude a contribution from intrinsic properties, understanding the specific contributions of different cell types, such as interneurons, which have distinct intrinsic properties and are known to play a crucial role in hippocampal function, remains an important avenue for future research that could build upon the findings of this study.

While this study offers valuable insights through its direct comparison across six regions, certain limitations suggest directions for future research. First, our analysis utilized a “neuron-by-neuron” classification approach, generating two distinct data points from each neuron to represent its averaged activity profile under “rewarded” and “nonrewarded” conditions. This method aimed to characterize the coding properties of individual neurons, treated as isolated samples, to uncover general trends within each region. Although this feature-based approach has proven informative for characterizing neural coding (Vivekanandhan et al., 2023) and enabled us to reveal the hierarchical distribution of reward information, information in these circuits is often encoded at the collective population level, incorporating neuronal correlations and interactions (Georgopoulos et al., 1986; Pouget et al., 2000; Averbeck et al., 2006; Mankin et al., 2012), or dynamically modulated on a trial-by-trial basis (Meyers et al., 2008). Therefore, crucial next steps involve applying simultaneous population decoding methods to capture synergistic interactions among neurons and extending this to “trial-by-trial” analyses to reveal transient network states that are not apparent from the averaged activity of individual units.

Second, models' performance and their interpretations are somewhat constrained by the feature set used. Although our models worked well, future work could investigate the impact of incorporating different sets of neural features. Expanding the feature space beyond the current set—for instance, by including measures of population synchrony, local field potential characteristics, metrics of temporal complexity such as fractal dimensions (Mehrabbeik et al., 2023; Vivekanandhan et al., 2023), or metrics specifically designed to quantify inhibition—could reveal complementary aspects of reward coding. In particular, features characterizing suppressive dynamics might aid significantly in interpreting the coding strategy of regions like vCA1 (see Results).

Third, it is also worth exploring alternative machine learning architectures, which could provide further insights for the reward representation. For example, deep neural networks or models explicitly designed for time-series analysis (like recurrent neural networks or long short-term memories) might capture complex temporal dependencies or nonlinear relationships in ways complementary to tree-based methods, potentially offering different perspectives on the underlying neural code, even if overall predictive accuracy is similar.

Finally, the observed hierarchy (vCA1/dCA1 > LEC/PPC > M1/M2) reflects the robustness of reward-related representation within the specific context of our self-initiated task. To understand the generality and flexibility of these representations, future studies should investigate how coding and regional contributions change under different behavioral conditions, such as tasks involving probabilistic rewards, explicit cues, or varying cognitive demands, allowing better comparison with findings from different paradigms. It is also important to remember that higher predictive accuracy indicates a strong correlation between regional activity and outcome, but does not necessarily equate to a greater causal role in the task.

It is also noteworthy that our task design introduces potential limitations regarding the interpretation of the neural signals. The deterministic reward schedule likely induced reward expectation signals that are intertwined with outcome-specific responses, which may explain the pre-action differentiation of neural activity in vCA1 (Fig. 1*D*). Furthermore, the sensory modalities associated with the outcomes differed: a gustatory reward versus an auditory error cue. Consequently, our machine learning models may have utilized sensory-specific features to distinguish between trial types. However, we argue that this reflects the naturalistic challenge the brain solves, as these sensory and expectation-related signals are integral components of the “reward-related information” in our task. While this approach is valid for assessing the overall representation of trial outcome, we acknowledge that this design does not isolate an abstract “value” signal. Future studies using probabilistic rewards or modality-controlled cues would be necessary to dissociate these distinct components of reward processing.

### Concluding statement

In conclusion, by applying a uniform machine learning framework across six brain regions, our primary finding is the demonstration of a robust functional hierarchy in reward-related information representation. This hierarchy is characterized by a progressive increase in predictive power from the motor cortices through the parahippocampal and parietal regions to the hippocampus. Building on this quantitative foundation, interpretable machine learning further provided qualitative insights, revealing a transformation in coding strategies: the information evolves from a graded, distributed code with higher uncertainty in the neocortex to a categorical, sharply binarized representation in the hippocampus. At this hierarchical apex, distinct coding strategies emerged: dCA1 relies primarily on precise spike timing, whereas vCA1 employs a multifaceted strategy integrating timing with firing rate suppression. These findings underscore the importance of considering the entire processing pipeline—from the distributed/uncertain codes in the neocortex to the specialized/integrated codes in the hippocampus—to fully understand how reward information is transformed to guide behavior.

## Synthesis

Reviewing Editor: Mihaela Iordanova, Concordia University - Loyola Campus Decisions are customarily a result of the Reviewing Editor and the peer reviewers coming together and discussing their recommendations until a consensus is reached. When revisions are invited, a fact-based synthesis statement explaining their decision and outlining what is needed to prepare a revision will be listed below. The following reviewer(s) agreed to reveal their identity: NONE. Note: If this manuscript was transferred from JNeurosci and a decision was made to accept the manuscript without peer review, a synthesis may not be available.

The reviewer and editor greatly enjoyed reading and reviewing this manuscript. They found the work valuable, making important contributions to the behavioral neurophysiology and computational neuroscience literature. He authors are commended on their Herculean technical achievement. The paper will be of high interest to several fields, spanning behavior to neural computation. Outside of the aforementioned elaboration on statistics, addressing the below additions, modifications, and suggestions would greatly improve the manuscript:

- the results section needs to be better balanced. In its present form it is to overly focused on the CA1 regions but this comes at a cost of looking past the bigger picture. Given that a real strength of the paper is the multiple region recording, this should be reflected in the results much more prominently.

- Table 1 reports just the top 6 classifiers for each area. It is recommended to show the bottom 6 classifiers or the bottom 6 that still exceed chance. Knowing whether the best 6 in M2 exceeded (or not) the worst 6 in CA1 might be really informative. It is not clear whether there is a clear break in performance after the top 6, or even comparing best to worst. That function could be really different across areas, which could indicate whatever features all the classifiers are picking up on could be really different across areas.

- In figure 1A, some schematic representation of the time windows used for analysis would be helpful. Something like dashed outlines overlaid on the trial structure schematic

- Something like the above would also help in figure 1B, in addition to elaboration on which trials were analyzed. All of them, just those right after a block switch, just before a block switch, in the middle? This could have a big effect on how the data should be interpreted.

- It would also be helpful to have some descriptive statistics on the trial numbers, number of units, and units per session analyzed. Classification analyses on 500 vs 1,000 neurons from the same area could have very different performance, and often the number of simultaneously recorded units correlates best with classifier performance. Similarly leave-out validation will perform much better with 400 trials than with 40, so it would help to know all this information, even if it was not all explicitly controlled for.

- please add “firing rate” to the y-axis in figure 1D

- Some discussion of why one kind of classifier might perform better or worse is warranted. Could it be the case that the findings here are just task dependent? For example, maybe support vector classifiers work well in a binary discrimination like the task here, but a discriminant classifier performs better for multiclass?

- Additional clarification of the discussion in lines 774-781 in needed as it is hard to understand in its current form. Is classifying from single neuron features taken from many neurons not a population decoding analysis?

- The reviewer greatly appreciated the discussion of the caveat that rewarded/nonrewarded trials are confounded by reward/sensory experience (auditory error signal) in lines 809-815. It is an important caveat and well discussed. Extending this a bit further, they wondered whether the effects hold true at other trial epochs. They were most interested in the pre-outcome period - this is often when very interesting predictive neural activity occurs. Incidentally, analyzing this epoch would also help address the reward/sensory confound. It is recognized that there are concerns about movement artifact (lines 320-324), so maybe this was not possible?

- Was there a low-cut filter applied to the spike band? I see 0.5 Hz to 10 kHz. It is typical to cut low frequencies at 100-300 Hz, especially if there are concerns about movement artifact and other noise.

- please double check equation 1 - formula for Cv, seems to be missing ‘i’ in the denominator.
