# Supplementary material for: Hierarchical Distribution of Reward Representation in the Cortical and Hippocampal Regions
Source: eNeuro. 2026 Feb 10;13(2):ENEURO.0256-25.2026. doi: 10.1523/ENEURO.0256-25.2026 (PMC12931971; doi:10.1523/ENEURO.0256-25.2026)
Supplement: Figure 2-1 — List of the 88 features derived from neuronal activity used for the machine learning classification. These features are grouped into three main categories: fundamental spiking properties, task-related spike-timing properties, and task-related firing-rate properties. Fundamental properties characterize a neuron's intrinsic firing patterns and waveform, using metrics like the coefficient of variation (Cv), local variation (Lv), spike width, and autocorrelogram (ACG) bias. Task-related features quantify neural dynamics around specific events. Spike-timing properties describe the statistical distribution of spike times, including their mean, standard deviation, skewness, kurtosis, and quartiles. Firing-rate properties are calculated from Peri-Event Time Histograms (PETHs) and include mean and peak firing rates in various windows, as well as a firing rate change (FRc) index. These task-dependent features were computed separately for events related to action (A) or outcome (O) and for trials contralateral (C) or ipsilateral (I) to the recording site. Download Figure 2-1, DOCX file. [file eneuro-13-ENEURO.0256-25.2026-s002.docx]

**Extended Data Figure 2-1**

*Feature names*

| Abbreviation | Explanation |
| --- | --- |
| Ongoing activity | Ground averaged spiking activity |
| Cv | Conventional coefficient of variation (Equation 1) ([Shinomoto et al., PLoS Comput Biol 2009](https://journals.plos.org/ploscompbiol/article?id=10.1371/journal.pcbi.1000433)) |
| Lv | A metric of local variation (Equation 2) ([Shinomoto et al., PLoS Biology 2009](https://journals.plos.org/ploscompbiol/article?id=10.1371/journal.pcbi.1000433)) |
| ACG bias | Classical ACG bias (median at 0–100 ms) ([Saiki et al., PLoS One 2014](https://journals.plos.org/plosone/article?id=10.1371/journal.pone.0098662)) |
| Peak ACG bias | Peak ACG bias (median at 0–50 ms) ([Saiki et al., Cereb Cortex 2018](https://academic.oup.com/cercor/article/28/3/1024/2964651)) |
| Baseline ACG bias | Baseline ACG bias (median at 50–250 ms) ([Saiki et al., Cereb Cortex 2018](https://academic.oup.com/cercor/article/28/3/1024/2964651)) |
| Spike duration | Time from spike onset to the first positive peak ([Isomura et al., Nat Neurosci 2009](https://www.nature.com/articles/nn.2431)) |
| Spike width | The elapsed time above the half amplitude of the positive spike waveform ([Isomura et al., Nat Neurosci 2009](https://www.nature.com/articles/nn.2431)) |
| Mean spike timing (OC) | Mean spike timing position in the 0 to 500 ms period of outcome-triggered raster plot in contralateral trials |
| SD of spike timing (OC) | Standard deviation of spike timing position in the 0 to 500 ms period of outcome-triggered raster plot in contralateral trials |
| Spike timing kurtosis (OC) | Kurtosis (degree of tailedness) of spike timing distribution in the 0 to 500 ms period of outcome-triggered raster plot in contralateral trials |
| Spike timing skewness (OC) | Skewness (asymmetry) of spike timing distribution in the 0 to 500 ms period of outcome-triggered raster plot in contralateral trials |
| Q1 spike timing (OC) | First quartile (Q1, 25%) of spike timing position in the 0 to 500 ms period of outcome-triggered raster plot in contralateral trials |
| Q2 spike timing (OC) | Second quartile (Q2, 50%, median) of spike timing position in the 0 to 500 ms period of outcome-triggered raster plot in contralateral trials |
| Q3 spike timing (OC) | Third quartile (Q3, 75%) of spike timing position in the 0 to 500 ms period of outcome-triggered raster plot in contralateral trials |
| KS statistic (OC) | KS statistic obtained from outcome-triggered rater plot in contralateral trials |
| Mean Spikes (OC) | The mean trial-by-trial spike count in the outcome-triggered raster for contralateral trials |
| Mean FR in 0–500 ms (OC) | Averaging firing rate in the 0 to 500 ms period of outcome-triggered PETH in contralateral trials |
| Mean FR in 0–50 ms (OC) | Averaging firing rate in the 0 to 50 ms period of outcome-triggered PETH in contralateral trials |
| Mean FR in 50–100 ms (OC) | Averaging firing rate in the 50 to 100 ms period of outcome-triggered PETH in contralateral trials |
| Mean FR in 100–250 ms (OC) | Averaging firing rate in the 100 to 250 ms period of outcome-triggered PETH in contralateral trials |
| Mean FR in 250–500 ms (OC) | Averaging firing rate in the 250 to 500 ms period of outcome-triggered PETH in contralateral trials |
| Peak FR in 0–500 ms (OC) | Peak firing rate in the 0 to 500 ms period of outcome-triggered PETH in contralateral trials |
| Peak FR in 0–50 ms (OC) | Peak firing rate in the 0 to 50 ms period of outcome-triggered PETH in contralateral trials |
| Peak FR in 50–100 ms (OC) | Peak firing rate in the 50 to 100 ms period of outcome-triggered PETH in contralateral trials |
| Peak FR in 100–250 ms (OC) | Peak firing rate in the 100 to 250 ms period of outcome-triggered PETH in contralateral trials |
| Peak FR in 250–500 ms (OC) | Peak firing rate in the 250 to 500 ms period of outcome-triggered PETH in contralateral trials |
| FRc index (OC) | FRc index obtained from outcome-triggered PETH in contralateral trials (Equation 3) |
| Mean spike timing (OI) | Mean spike timing position in the 0 to 500 ms period of outcome-triggered raster plot in ipsilateral trials |
| SD of spike timing (OI) | Standard deviation of spike timing position in the 0 to 500 ms period of outcome-triggered raster plot in ipsilateral trials |
| Spike timing kurtosis (OI) | Kurtosis (degree of tailedness) of spike timing distribution in the 0 to 500 ms period of outcome-triggered raster plot in ipsilateral trials |
| Spike timing skewness (OI) | Skewness (asymmetry) of spike timing distribution in the 0 to 500 ms period of outcome-triggered raster plot in ipsilateral trials |
| Q1 spike timing (OI) | First quartile (Q1, 25%) of spike timing position in the 0 to 500 ms period of outcome-triggered raster plot in ipsilateral trials |
| Q2 spike timing (OI) | Second quartile (Q2, 50%, median) of spike timing position in the 0 to 500 ms period of outcome-triggered raster plot in ipsilateral trials |
| Q3 spike timing (OI) | Third quartile (Q3, 75%) of spike timing position in the 0 to 500 ms period of outcome-triggered raster plot in ipsilateral trials |
| KS statistic (OI) | KS statistic obtained from outcome-triggered rater plot in ipsilateral trials |
| Mean Spikes (OI) | The mean trial-by-trial spike count in the outcome-triggered raster for ipsilateral trials |
| Mean FR in 0–500 ms (OI) | Averaging firing rate in the 0 to 500 ms period of outcome-triggered PETH in ipsilateral trials |
| Mean FR in 0–50 ms (OI) | Averaging firing rate in the 0 to 50 ms period of outcome-triggered PETH in ipsilateral trials |
| Mean FR in 50–100 ms (OI) | Averaging firing rate in the 50 to 100 ms period of outcome-triggered PETH in ipsilateral trials |
| Mean FR in 100–250 ms (OI) | Averaging firing rate in the 100 to 250 ms period of outcome-triggered PETH in ipsilateral trials |
| Mean FR in 250–500 ms (OI) | Averaging firing rate in the 250 to 500 ms period of outcome-triggered PETH in ipsilateral trials |
| Peak FR in 0–500 ms (OI) | Peak firing rate in the 0 to 500 ms period of outcome-triggered PETH in ipsilateral trials |
| Peak FR in 0–50 ms (OI) | Peak firing rate in the 0 to 50 ms period of outcome-triggered PETH in ipsilateral trials |
| Peak FR in 50–100 ms (OI) | Peak firing rate in the 50 to100 ms period of outcome-triggered PETH in ipsilateral trials |
| Peak FR in 100–250 ms (OI) | Peak firing rate in the 100 to 250 ms period of outcome-triggered PETH in ipsilateral trials |
| Peak FR in 250–500 ms (OI) | Peak firing rate in the 250 to 500 ms period of outcome-triggered PETH in ipsilateral trials |
| FRc index (OI) | FRc index obtained from outcome-triggered PETH in ipsilateral trials (Equation 3) |
| Mean spike timing (AC) | Mean spike timing position in the −500 to 0 ms period of action-triggered raster plot in contralateral trials |
| SD of spike timing (AC) | Standard deviation of spike timing position in the −500 to 0 ms period of action-triggered raster plot in contralateral trials |
| Spike timing kurtosis (AC) | Kurtosis (degree of tailedness) of spike timing distribution in the −500 to 0 ms period of action-triggered raster plot in contralateral trials |
| Spike timing skewness (AC) | Skewness (asymmetry) of spike timing distribution in the −500 to 0 ms period of action-triggered raster plot in contralateral trials |
| Q1 spike timing (AC) | First quartile (Q1, 25%) of spike timing position in the −500 to 0 ms period of action-triggered raster plot in contralateral trials |
| Q2 spike timing (AC) | Second quartile (Q2, 50%, median) of spike timing position in the −500 to 0 ms period of action-triggered raster plot in contralateral trials |
| Q3 spike timing (AC) | Third quartile (Q3, 75%) of spike timing position in the −500 to 0 ms period of action-triggered raster plot in contralateral trials |
| KS statistic (AC) | KS statistic obtained from action-triggered rater plot in contralateral trials |
| Mean Spikes (AC) | The mean trial-by-trial spike count in the action-triggered raster for contralateral trials |
| Mean FR in −500 to 0 ms (AC) | Averaging firing rate in the −500 to 0 ms period of action-triggered PETH in contralateral trials |
| Mean FR in −500 to -250 ms (AC) | Averaging firing rate in the −500 to −250 ms period of action-triggered PETH in contralateral trials |
| Mean FR in −250 to -100 ms (AC) | Averaging firing rate in the −250 to −100 ms period of action-triggered PETH in contralateral trials |
| Mean FR in −100 to -50 ms (AC) | Averaging firing rate in the −100 to −50 ms period of action-triggered PETH in contralateral trials |
| Mean FR in −50 to 0 ms (AC) | Averaging firing rate in the −50 to 0 ms period of action-triggered PETH in contralateral trials |
| Peak FR in −500 to 0 ms (AC) | Peak firing rate in the −500 to 0 ms period of action-triggered PETH in contralateral trials |
| Peak FR in −500 to −250 ms (AC) | Peak firing rate in the −500 to −200 ms period of action-triggered PETH in contralateral trials |
| Peak FR in −250 to −100 ms (AC) | Peak firing rate in the −250 to −100 ms period of action-triggered PETH in contralateral trials |
| Peak FR in −100 to −50 ms (AC) | Peak firing rate in the −100 to −50 ms period of action-triggered PETH in contralateral trials |
| Peak FR in −50 to 0 ms (AC) | Peak firing rate in the −50 to 0 ms period of action-triggered PETH in contralateral trials |
| FRc index (AC) | FRc index obtained from action-triggered PETH in contralateral trials (Equation 3) |
| Mean spike timing (AI) | Mean spike timing position in the −500 to 0 ms period of action-triggered raster plot in ipsilateral trials |
| SD of spike timing (AI) | Standard deviation of spike timing position in the −500 to 0 ms period of action-triggered raster plot in ipsilateral trials |
| Spike timing kurtosis (AI) | Kurtosis (degree of tailedness) of spike timing distribution in the −500 to 0 ms period of action-triggered raster plot in ipsilateral trials |
| Spike timing skewness (AI) | Skewness (asymmetry) of spike timing distribution in the −500 to 0 ms period of action-triggered raster plot in ipsilateral trials |
| Q1 spike timing (AI) | First quartile (Q1, 25%) of spike timing position in the −500 to 0 ms period of action-triggered raster plot in ipsilateral trials |
| Q2 spike timing (AI) | Second quartile (Q2, 50%, median) of spike timing position in the −500 to 0 ms period of action-triggered raster plot in ipsilateral trials |
| Q3 spike timing (AI) | Third quartile (Q3, 75%) of spike timing position in the −500 to 0 ms period of action-triggered raster plot in ipsilateral trials |
| KS statistic (AI) | KS statistic obtained from action-triggered rater plot in ipsilateral trials |
| Mean Spikes (AI) | The mean trial-by-trial spike count in the action-triggered raster for ipsilateral trials |
| Mean FR in 0–500 ms (AI) | Averaging firing rate in the 0 to 500 ms period of action-triggered PETH in ipsilateral trials |
| Mean FR in 0–50 ms (AI) | Averaging firing rate in the 0 to 50 ms period of action-triggered PETH in ipsilateral trials |
| Mean FR in 50–100 ms (AI) | Averaging firing rate in the 50 to 100 ms period of action-triggered PETH in ipsilateral trials |
| Mean FR in 100–250 ms (AI) | Averaging firing rate in the 100 to 250 ms period of action-triggered PETH in ipsilateral trials |
| Mean FR in 250–500 ms (AI) | Averaging firing rate in the 250 to 500 ms period of action-triggered PETH in ipsilateral trials |
| Peak FR in 0–500 ms (AI) | Peak firing rate in the 0 to 500 ms period of action-triggered PETH in ipsilateral trials |
| Peak FR in 0–50 ms (AI) | Peak firing rate in the 0 to 50 ms period of action-triggered PETH in ipsilateral trials |
| Peak FR in 50–100 ms (AI) | Peak firing rate in the 50 to 100 ms period of action-triggered PETH in ipsilateral trials |
| Peak FR in 100–250 ms (AI) | Peak firing rate in the 100 to 250 ms period of action-triggered PETH in ipsilateral trials |
| Peak FR in 250–500 ms (AI) | Peak firing rate in the 250 to 500 ms period of action-triggered PETH in ipsilateral trials |
| FRc index (AI) | FRc index obtained from action-triggered PETH in ipsilateral trials (Equation 3) |

**Extended Data Figure 2-1.** List of the 88 features derived from neuronal activity used for the machine learning classification. These features are grouped into three main categories: fundamental spiking properties, task-related spike-timing properties, and task-related firing-rate properties. Fundamental properties characterize a neuron's intrinsic firing patterns and waveform, using metrics like the coefficient of variation (Cv), local variation (Lv), spike width, and autocorrelogram (ACG) bias. Task-related features quantify neural dynamics around specific events. Spike-timing properties describe the statistical distribution of spike times, including their mean, standard deviation, skewness, kurtosis, and quartiles. Firing-rate properties are calculated from Peri-Event Time Histograms (PETHs) and include mean and peak firing rates in various windows, as well as a firing rate change (FRc) index. These task-dependent features were computed separately for events related to action (A) or outcome (O) and for trials contralateral (C) or ipsilateral (I) to the recording site.
