# Supplementary material for: Hierarchical Distribution of Reward Representation in the Cortical and Hippocampal Regions
Source: eNeuro. 2026 Feb 10;13(2):ENEURO.0256-25.2026. doi: 10.1523/ENEURO.0256-25.2026 (PMC12931971; doi:10.1523/ENEURO.0256-25.2026)
Supplement: Figure 4-1 — Summary table of the two-way analysis of variance (ANOVA) performed to assess the effects of Brain Region and Model Architecture on classification accuracy. The analysis treated individual test accuracy scores from three independent training/testing repetitions as observations (N = 54). Factors: Brain Region (6 levels: M1, M2, PPC, LEC, vCA1, dCA1) and Model Architecture (3 levels: CatBoost, LightGBM, XGBoost). Results: A highly significant main effect of Brain Region was observed (F(5,36) = 59.16, p < 0.0001), confirming the presence of a statistically distinct performance gradient across regions. In contrast, neither the main effect of Model Architecture (F(2,36) = 0.57, p = 0.57) nor the interaction between Region and Architecture (F(10,36) = 0.19, p = 0.99) was significant. These results statistically demonstrate that the observed functional hierarchy (Hippocampus > Parahippocampal/Parietal > Motor) is a robust biological property that persists regardless of the specific machine learning algorithm used. Download Figure 4-1, DOCX file. [file eneuro-13-ENEURO.0256-25.2026-s003.docx]

**Extended Data Figure 4-1**

*Statistical validation of performance hierarchy via two-way ANOVA.*

|  | Sum of Saure | Degree of freedom | F-value | PR (>F) |
| --- | --- | --- | --- | --- |
| Brain Region | 0.2600 | 5.0 | 59.1579 | <0.0001 |
| Model Architecture | 0.0010 | 2.0 | 0.5663 | 0.5726 |
| Interaction  (Region x Architecture) | 00017 | 10.0 | 0.1883 | 0.9961 |
| Residual | 0.0316 | 36.0 |  |  |

**Extended Data Figure 4-1.** Summary table of the two-way analysis of variance (ANOVA) performed to assess the effects of Brain Region and Model Architecture on classification accuracy. The analysis treated individual test accuracy scores from three independent training/testing repetitions as observations (N = 54). Factors: Brain Region (6 levels: M1, M2, PPC, LEC, vCA1, dCA1) and Model Architecture (3 levels: CatBoost, LightGBM, XGBoost). Results: A highly significant main effect of Brain Region was observed (*F*_(5,36)_ = 59.16, *p* < 0.0001), confirming the presence of a statistically distinct performance gradient across regions. In contrast, neither the main effect of Model Architecture (*F*_(2,36)_ = 0.57, *p* = 0.57) nor the interaction between Region and Architecture (*F*_(10,36)_ = 0.19, *p* = 0.99) was significant. These results statistically demonstrate that the observed functional hierarchy (Hippocampus > Parahippocampal/Parietal > Motor) is a robust biological property that persists regardless of the specific machine learning algorithm used**.**
