# Supplementary material for: Hierarchical Distribution of Reward Representation in the Cortical and Hippocampal Regions
Source: eNeuro. 2026 Feb 10;13(2):ENEURO.0256-25.2026. doi: 10.1523/ENEURO.0256-25.2026 (PMC12931971; doi:10.1523/ENEURO.0256-25.2026)
Supplement: Figure 4-2 — The table summarizes the statistical validation of the hyperparameter-tuned models, comparing their actual classification accuracy (“True accuracy”) against chance-level performance distributions derived from shuffle control analyses (“Permutation accuracy”). For each of the 18 conditions (comprising 6 brain regions and 3 model architectures), trial outcome labels were randomly shuffled 1,000 times to generate a null distribution. “True accuracy” denotes the model's performance on the original test set, while “Permutation accuracy” reports the mean and standard deviation (SD) of accuracy scores from the shuffled datasets. Statistical significance (p-value) was determined with Bonferroni correction for multiple comparisons (correction factor = 18). A p-value of 0.0180 represents the resolution limit of this permutation test (1/1,000 iterations × 18 comparisons), indicating that the true accuracy surpassed the accuracy of all 1,000 permuted samples (uncorrected p < 0.001) in every instance. These results confirm that classification performance was significantly above chance across all regions and models. Download Figure 4-2, DOCX file. [file eneuro-13-ENEURO.0256-25.2026-s004.docx]

**Extended Data Figure 4-2**

*Permutation test results validating classification performance against chance.*

| ROI | Model Architecture | True accuracy | Permutation accuracy (mean) | Permutation accuracy  (SD) | p-value |
| --- | --- | --- | --- | --- | --- |
| M1 | CatBoost | 0.6408 | 0.4995 | 0.0127 | 0.0180 |
|  | LightGBM | 0.6390 | 0.4998 | 0.0129 | 0.0180 |
|  | XGBosot | 0.6310 | 0.5006 | 0.0128 | 0.0180 |
| M2 | CatBoost | 0.6524 | 0.5010 | 0.0105 | 0.0180 |
|  | LightGBM | 0.6569 | 0.5005 | 0.0106 | 0.0180 |
|  | XGBosot | 0.6369 | 0.5007 | 0.0108 | 0.0180 |
| PPC | CatBoost | 0.6933 | 0.5009 | 0.0144 | 0.0180 |
|  | LightGBM | 0.6684 | 0.5008 | 0.0140 | 0.0180 |
|  | XGBosot | 0.6821 | 0.5020 | 0.0148 | 0.0180 |
| LEC | CatBoost | 0.7704 | 0.5008 | 0.0105 | 0.0180 |
|  | LightGBM | 0.7618 | 0.5013 | 0.0103 | 0.0180 |
|  | XGBosot | 0.7573 | 0.5016 | 0.0104 | 0.0180 |
| vCA1 | CatBoost | 0.8027 | 0.5084 | 0.0122 | 0.0180 |
|  | LightGBM | 0.8121 | 0.5117 | 0.0120 | 0.0180 |
|  | XGBosot | 0.7984 | 0.5096 | 0.0120 | 0.0180 |
| dCA1 | CatBoost | 0.8083 | 0.5037 | 0.0153 | 0.0180 |
|  | LightGBM | 0.7935 | 0.5029 | 0.0153 | 0.0180 |
|  | XGBosot | 0.7994 | 0.5039 | 0.0157 | 0.0180 |

**Extended Data Figure 4-2.** The table summarizes the statistical validation of the hyperparameter-tuned models, comparing their actual classification accuracy ("True accuracy") against chance-level performance distributions derived from shuffle control analyses ("Permutation accuracy"). For each of the 18 conditions (comprising 6 brain regions and 3 model architectures), trial outcome labels were randomly shuffled 1,000 times to generate a null distribution. "True accuracy" denotes the model's performance on the original test set, while "Permutation accuracy" reports the mean and standard deviation (SD) of accuracy scores from the shuffled datasets. Statistical significance (p-value) was determined with Bonferroni correction for multiple comparisons (correction factor = 18). A p-value of 0.0180 represents the resolution limit of this permutation test (1/1,000 iterations × 18 comparisons), indicating that the true accuracy surpassed the accuracy of all 1,000 permuted samples (uncorrected *p* < 0.001) in every instance. These results confirm that classification performance was significantly above chance across all regions and models.
