# Supplementary material for: Hierarchical Distribution of Reward Representation in the Cortical and Hippocampal Regions
Source: eNeuro. 2026 Feb 10;13(2):ENEURO.0256-25.2026. doi: 10.1523/ENEURO.0256-25.2026 (PMC12931971; doi:10.1523/ENEURO.0256-25.2026)
Supplement: Figure 5-1 — This table summarizes the classification performance and the top-ranking features for the best model architecture (CatBoost) in the dorsal CA1 (dCA1) region across three independent training/testing repetitions (Repeat 0, 1, and 2). The best model architecture was determined based on the highest mean accuracy across repetitions (see Materials and Methods). For each repetition, the table lists the performance metrics (Accuracy and AUC) on the held-out test set, with the maximum values across repetitions indicated by asterisks (*). The top 9 features with the highest mean absolute SHAP values are listed in descending order of importance. Features that consistently ranked within the top 9 across all three repetitions are highlighted in bold text. This consistency underscores the robust contribution of specific spike timing distribution features (e.g., skewness, quartiles) to the model's predictions in dCA1, confirming that these coding strategies are stable biological properties rather than artifacts of specific data splits or model initializations. Download Figure 5-1, DOCX file. [file eneuro-13-ENEURO.0256-25.2026-s005.docx]

**Extended Data Figure 5-1**

*Model performance and top-contributing features across independent repetitions for dCA1*

| Repeat | | 0 | 1 | 2 |
| --- | --- | --- | --- | --- |
| Accuracy | | 0.8348 * | 0.7965 | 0.7935 |
| AUC | | 0.9162 * | 0.8889 | 0.8734 |
| Top Features | 1 | **Spike timing skewness (OC)** | **Spike timing skewness (OC)** | **Spike timing skewness (OI)** |
|  | 2 | Q1 spike timing (OI) | Q1 spike timing (OI) | **Spike timing skewness (OC)** |
|  | 3 | Q3 spike timing (OI) | Q3 spike timing (OI) | Spike timing skewness (AI) |
|  | 4 | KS statistic (AI) | **Spike timing skewness (OI)** | KS statistic (AI) |
|  | 5 | **Spike timing skewness (OI)** | Spike timing skewness (AI) | Q3 spike timing (OI) |
|  | 6 | **Mean FR in 50–100 ms (OI)** | **Spike timing kurtosis (OC)** | **Spike timing kurtosis (OC)** |
|  | 7 | Spike timing skewness (AI) | KS statistic (AI) | **Mean FR in 50–100 ms (OI)** |
|  | 8 | **Spike timing kurtosis (OC)** | **Mean FR in 50–100 ms (OI)** | Spike timing skewness (AC) |
|  | 9 | Spike timing skewness (AC) | SD of spike timing (AC) | Spike timing kurtosis (AC) |

**Extended Data Figure 5-1.** This table summarizes the classification performance and the top-ranking features for the best model architecture (CatBoost) in the dorsal CA1 (dCA1) region across three independent training/testing repetitions (Repeat 0, 1, and 2). The best model architecture was determined based on the highest mean accuracy across repetitions (see Materials and Methods). For each repetition, the table lists the performance metrics (Accuracy and AUC) on the held-out test set, with the maximum values across repetitions indicated by asterisks (*). The top 9 features with the highest mean absolute SHAP values are listed in descending order of importance. Features that consistently ranked within the top 9 across all three repetitions are highlighted in bold text. This consistency underscores the robust contribution of specific spike timing distribution features (e.g., skewness, quartiles) to the model's predictions in dCA1, confirming that these coding strategies are stable biological properties rather than artifacts of specific data splits or model initializations.
