# Supplementary material for: Hierarchical Distribution of Reward Representation in the Cortical and Hippocampal Regions
Source: eNeuro. 2026 Feb 10;13(2):ENEURO.0256-25.2026. doi: 10.1523/ENEURO.0256-25.2026 (PMC12931971; doi:10.1523/ENEURO.0256-25.2026)
Supplement: Figure 6-1 — This table summarizes the classification performance and the top-ranking features for the best model architecture (LightGBM) in the ventral CA1 (vCA1) region across three independent training/testing repetitions (Repeat 0, 1, and 2). The best model architecture was determined based on the highest mean accuracy across repetitions (see Materials and Methods). For each repetition, the table lists the performance metrics (Accuracy and AUC) on the held-out test set, with the maximum values across repetitions indicated by asterisks (*). The top 9 features with the highest mean absolute SHAP values are listed in descending order of importance. Features that consistently ranked within the top 9 across all three repetitions are highlighted in bold text, indicating the robust contribution of specific action- and outcome-related statistics (e.g., KS statistics). Notably, although not present in all three repetitions, the first quartile of post-reward spike timing (Q1 spike timing (OC)) ranked highly in two out of three repetitions (Repeat 0 and 2), including the best-performing instance (Repeat 0), further supporting the relevance of temporal coding features in this region. Download Figure 6-1, DOCX file. [file eneuro-13-ENEURO.0256-25.2026-s006.docx]

**Extended Data Figure 6-1**

*Model performance and top-contributing features across independent repetitions for vCA1*

| Repeat | | 0 | 1 | 2 |
| --- | --- | --- | --- | --- |
| Accuracy | | 0.8315 * | 0.7753 | 0.8296 |
| AUC | | 0.9022 * | 0.8427 | 0.8977 |
| Top Features | 1 | Mean FR in −50 to 0 ms (AC) | Mean FR in −100 to -50 ms (AC) | Mean FR in −100 to -50 ms (AC) |
|  | 2 | **KS statistic (AI)** | **KS statistic (AI)** | **KS statistic (AC)** |
|  | 3 | **KS statistic (AC)** | **KS statistic (AC)** | **KS statistic (AI)** |
|  | 4 | Q1 spike timing (OC) | FRc index (AC) | FRc index (AC) |
|  | 5 | SD of spike timing (AI) | SD of spike timing (AI) | **KS statistic (OI)** |
|  | 6 | **KS statistic (OI)** | **KS statistic (OI)** | Q1 spike timing (OC) |
|  | 7 | FRc index (OC) | Baseline ACG bias | Q3 spike timing (AI) |
|  | 8 | FRc index (AC) | Q3 spike timing (OC) | SD of spike timing (AI) |
|  | 9 | KS statistic (OC) | Q3 spike timing (AI) | Baseline ACG bias |

**Extended Data Figure 6-1.** This table summarizes the classification performance and the top-ranking features for the best model architecture (LightGBM) in the ventral CA1 (vCA1) region across three independent training/testing repetitions (Repeat 0, 1, and 2). The best model architecture was determined based on the highest mean accuracy across repetitions (see Materials and Methods). For each repetition, the table lists the performance metrics (Accuracy and AUC) on the held-out test set, with the maximum values across repetitions indicated by asterisks (*). The top 9 features with the highest mean absolute SHAP values are listed in descending order of importance. Features that consistently ranked within the top 9 across all three repetitions are highlighted in bold text, indicating the robust contribution of specific action- and outcome-related statistics (e.g., KS statistics). Notably, although not present in all three repetitions, the first quartile of post-reward spike timing (Q1 spike timing (OC)) ranked highly in two out of three repetitions (Repeat 0 and 2), including the best-performing instance (Repeat 0), further supporting the relevance of temporal coding features in this region.
