# Supplementary material for: Hierarchical Distribution of Reward Representation in the Cortical and Hippocampal Regions
Source: eNeuro. 2026 Feb 10;13(2):ENEURO.0256-25.2026. doi: 10.1523/ENEURO.0256-25.2026 (PMC12931971; doi:10.1523/ENEURO.0256-25.2026)
Supplement: Figure 7-1 — This table summarizes the classification performance and the top-ranking features for the best model architecture (CatBoost) in the lateral entorhinal cortex (LEC) across three independent training/testing repetitions (Repeat 0, 1, and 2). The best model architecture was determined based on the highest mean accuracy across repetitions (see Materials and Methods). For each repetition, the table lists the performance metrics (Accuracy and AUC) on the held-out test set, with the maximum values across repetitions indicated by asterisks (*). The top 9 features with the highest mean absolute SHAP values are listed in descending order of importance. Features that consistently ranked within the top 9 across all three repetitions are highlighted in bold text. Notably, the FRc index (outcome-related firing rate change) consistently appeared as a top feature, reflecting the dominance of rate-based coding (suppression) in this region. Additionally, temporal features such as Q1 spike timing and KS statistic ranked within the top 9 in two out of three repetitions (including the best-performing Repeat 0), suggesting that while rate coding is primary, the LEC also exhibits emerging temporal coding properties, consistent with its role as a transitional gateway to the hippocampus. Download Figure 7-1, DOCX file. [file eneuro-13-ENEURO.0256-25.2026-s007.docx]

**Extended Data Figure 7-1**

*Model performance and top-contributing features across independent repetitions for LEC*

| Repeat | | 0 | 1 | 2 |
| --- | --- | --- | --- | --- |
| Accuracy | | 0.7808 * | 0.7659 | 0.7645 |
| AUC | | 0.8442 * | 0.8568 | 0.8407 |
| Top Features | 1 | KS statistic (AI) | Mean FR in 100–250 ms (OI) | Mean FR in −50 to 0 ms (AI) |
|  | 2 | Mean FR in 50–100 ms (OI) | KS statistic (AI) | KS statistic (AI) |
|  | 3 | Q2 spike timing (OC) | KS statistic (AC) | KS statistic (AC) |
|  | 4 | KS statistic (AC) | KS statistic (OI) | **FRc index (OC)** |
|  | 5 | **FRc index (OC)** | Q3 spike timing (OC) | Q3 spike timing (OC) |
|  | 6 | SD of spike timing (AC) | Spike timing kurtosis (OC) | Spike timing skewness (OC) |
|  | 7 | SD of spike timing (AI) | **FRc index (OC)** | KS statistic (OI) |
|  | 8 | SD of spike timing (OI) | SD of spike timing (OI) | SD of spike timing (AI) |
|  | 9 | Spike timing skewness (AI) | Q2 spike timing (OI) | Q2 spike timing (AI) |

**Extended Data Figure 7-1.** This table summarizes the classification performance and the top-ranking features for the best model architecture (CatBoost) in the lateral entorhinal cortex (LEC) across three independent training/testing repetitions (Repeat 0, 1, and 2). The best model architecture was determined based on the highest mean accuracy across repetitions (see Materials and Methods). For each repetition, the table lists the performance metrics (Accuracy and AUC) on the held-out test set, with the maximum values across repetitions indicated by asterisks (*). The top 9 features with the highest mean absolute SHAP values are listed in descending order of importance. Features that consistently ranked within the top 9 across all three repetitions are highlighted in bold text. Notably, the FRc index (outcome-related firing rate change) consistently appeared as a top feature, reflecting the dominance of rate-based coding (suppression) in this region. Additionally, temporal features such as Q1 spike timing and KS statistic ranked within the top 9 in two out of three repetitions (including the best-performing Repeat 0), suggesting that while rate coding is primary, the LEC also exhibits emerging temporal coding properties, consistent with its role as a transitional gateway to the hippocampus.
