# Supplementary material for: Hierarchical Distribution of Reward Representation in the Cortical and Hippocampal Regions
Source: eNeuro. 2026 Feb 10;13(2):ENEURO.0256-25.2026. doi: 10.1523/ENEURO.0256-25.2026 (PMC12931971; doi:10.1523/ENEURO.0256-25.2026)
Supplement: Figure 7-2 — This table summarizes the classification performance and the top-ranking features for the best model architecture (CatBoost) in the posterior parietal cortex (PPC) across three independent training/testing repetitions (Repeat 0, 1, and 2). The best model architecture was determined based on the highest mean accuracy across repetitions (see Materials and Methods). For each repetition, the table lists the performance metrics (Accuracy and AUC) on the held-out test set, with the maximum values across repetitions indicated by asterisks (*). The top features with the highest mean absolute SHAP values are listed in descending order of importance. Features that consistently ranked within the top list across all three repetitions are highlighted in bold text. Common features consistently identified include Mean Firing Rate in 0–50 ms, spike timing skewness (OI), KS statistic (OC), and Lv. Notably, the consistent contribution of Lv, a metric of intrinsic firing regularity, implies that the model relies not only on task-evoked responses but also on the intrinsic properties of neurons, suggesting that specific cell types with distinct firing characteristics play a significant role in encoding reward information in the PPC. Download Figure 7-2, DOCX file. [file eneuro-13-ENEURO.0256-25.2026-s008.docx]

**Extended Data Figure 7-2**

*Model performance and top-contributing features across independent repetitions for PPC*

| Repeat | | 0 | 1 | 2 |
| --- | --- | --- | --- | --- |
| Accuracy | | 0.7191 * | 0.7139 | 0.6469 |
| AUC | | 0.7765 * | 0.7810 | 0.7216 |
| Top Features | 1 | **Mean FR in 0–50 ms (OI)** | **Mean FR in 0–50 ms (OI)** | **Mean FR in 0–50 ms (OI)** |
|  | 2 | **Spike timing skewness (OI)** | **Spike timing skewness (OI)** | **Spike timing skewness (OI)** |
|  | 3 | KS statistic (AI) | KS statistic (AI) | KS statistic (AC) |
|  | 4 | **KS statistic (OC)** | KS statistic (AC) | **KS statistic (OC)** |
|  | 5 | KS statistic (AC) | **KS statistic (OC)** | KS statistic (AI) |
|  | 6 | **Lv** | Spike timing kurtosis (AC) | **Lv** |
|  | 7 | Spike timing kurtosis (AC) | **Lv** | SD of spike timing (AC) |
|  | 8 | Spike timing kurtosis (AI) | SD of spike timing (AI) | Spike timing kurtosis (OC) |
|  | 9 | SD of spike timing (AC) | SD of spike timing (AC) | SD of spike timing (AI) |

**Extended Data Figure 7-2.** This table summarizes the classification performance and the top-ranking features for the best model architecture (CatBoost) in the posterior parietal cortex (PPC) across three independent training/testing repetitions (Repeat 0, 1, and 2). The best model architecture was determined based on the highest mean accuracy across repetitions (see Materials and Methods). For each repetition, the table lists the performance metrics (Accuracy and AUC) on the held-out test set, with the maximum values across repetitions indicated by asterisks (*). The top features with the highest mean absolute SHAP values are listed in descending order of importance. Features that consistently ranked within the top list across all three repetitions are highlighted in bold text. Common features consistently identified include Mean Firing Rate in 0–50 ms, spike timing skewness (OI), KS statistic (OC), and Lv. Notably, the consistent contribution of Lv, a metric of intrinsic firing regularity, implies that the model relies not only on task-evoked responses but also on the intrinsic properties of neurons, suggesting that specific cell types with distinct firing characteristics play a significant role in encoding reward information in the PPC.
