# Supplementary material for: Hierarchical Distribution of Reward Representation in the Cortical and Hippocampal Regions
Source: eNeuro. 2026 Feb 10;13(2):ENEURO.0256-25.2026. doi: 10.1523/ENEURO.0256-25.2026 (PMC12931971; doi:10.1523/ENEURO.0256-25.2026)
Supplement: Figure 7-3 — This table summarizes the classification performance and the top-ranking features for the best model architecture (LightGBM) in the secondary motor cortex (M2) across three independent training/testing repetitions (Repeat 0, 1, and 2). The best model architecture was determined based on the highest mean accuracy across repetitions (see Materials and Methods). For each repetition, the table lists the performance metrics (Accuracy and AUC) on the held-out test set, with the maximum values across repetitions indicated by asterisks (*). The top 9 features with the highest mean absolute SHAP values are listed in descending order of importance. Features that consistently ranked within the top 9 across all three repetitions are highlighted in bold text. Common features consistently identified include KS statistic (OC,OI) and SD of spike timing (OC). The robust contribution of high-order statistical features like the KS statistic (quantifying distribution uniformity) and SD of spike timing, rather than simple firing rate magnitude, reflects the “subtle and distributed” coding strategy characteristic of M2. Note that the classification accuracy in M2 is markedly lower compared to the hippocampal (dCA1, vCA1) and parahippocampal (LEC) regions, supporting the functional hierarchy described in the main text. Download Figure 7-3, DOCX file. [file eneuro-13-ENEURO.0256-25.2026-s009.docx]

**Extended Data Figure 7-3**

*Model performance and top-contributing features across independent repetitions for M2*

| Repeat | | 0 | 1 | 2 |
| --- | --- | --- | --- | --- |
| Accuracy | | 0.6720 * | 0.6293 | 0.6693 |
| AUC | | 0.7235 * | 0.6740 | 0.7213 |
| Top Features | 1 | **KS statistic (OC)** | **KS statistic (OC)** | Mean FR in 100–250 ms (OC) |
|  | 2 | Mean FR in 100–250 ms (OC) | Mean FR in −50 to 0 ms (AI) | **KS statistic (OC)** |
|  | 3 | **KS statistic (OI)** | **KS statistic (OI)** | **KS statistic (OI)** |
|  | 4 | Q2 spike timing (OC) | **SD of spike timing (OC)** | **SD of spike timing (OC)** |
|  | 5 | **SD of spike timing (OC)** | Q2 spike timing (OC) | Q1 spike timing (OC) |
|  | 6 | Mean Spikes (OI) | Lv | Spike timing skewness (OI) |
|  | 7 | FRc index (AC) | FRc index (AC) | Spike timing kurtosis (AI) |
|  | 8 | KS statistic (AC) | SD of spike timing (AI) | KS statistic (AC) |
|  | 9 | Spike timing kurtosis (OI) | KS statistic (AI) | FRc index (AC) |

**Extended Data Figure 7-3.** This table summarizes the classification performance and the top-ranking features for the best model architecture (LightGBM) in the secondary motor cortex (M2) across three independent training/testing repetitions (Repeat 0, 1, and 2). The best model architecture was determined based on the highest mean accuracy across repetitions (see Materials and Methods). For each repetition, the table lists the performance metrics (Accuracy and AUC) on the held-out test set, with the maximum values across repetitions indicated by asterisks (*). The top 9 features with the highest mean absolute SHAP values are listed in descending order of importance. Features that consistently ranked within the top 9 across all three repetitions are highlighted in bold text. Common features consistently identified include KS statistic (OC,OI) and SD of spike timing (OC). The robust contribution of high-order statistical features like the KS statistic (quantifying distribution uniformity) and SD of spike timing, rather than simple firing rate magnitude, reflects the "subtle and distributed" coding strategy characteristic of M2. Note that the classification accuracy in M2 is markedly lower compared to the hippocampal (dCA1, vCA1) and parahippocampal (LEC) regions, supporting the functional hierarchy described in the main text
