# Supplementary material for: Hierarchical Distribution of Reward Representation in the Cortical and Hippocampal Regions
Source: eNeuro. 2026 Feb 10;13(2):ENEURO.0256-25.2026. doi: 10.1523/ENEURO.0256-25.2026 (PMC12931971; doi:10.1523/ENEURO.0256-25.2026)
Supplement: Figure 7-4 — This table summarizes the classification performance and the top-ranking features for the best model architecture (CatBoost) in the primary motor cortex (M1) across three independent training/testing repetitions (Repeat 0, 1, and 2). The best model architecture was determined based on the highest mean accuracy across repetitions (see Materials and Methods). For each repetition, the table lists the performance metrics (Accuracy and AUC) on the held-out test set, with the maximum values across repetitions indicated by asterisks (*). The top 9 features with the highest mean absolute SHAP values are listed in descending order of importance. Features that consistently ranked within the top 9 across all three repetitions are highlighted in bold text. Common features consistently identified include spike timing skewness (OC and OI) and KS statistic (OC and OI). Similar to M2, the classification accuracy in M1 is substantially lower than in the hippocampal and parahippocampal regions, consistent with its position at the lower end of the functional hierarchy. Download Figure 7-4, DOCX file. [file eneuro-13-ENEURO.0256-25.2026-s010.docx]

**Extended Data Figure 7-4**

*Model performance and top-contributing features across independent repetitions for M1*

| Repeat | | 0 | 1 | 2 |
| --- | --- | --- | --- | --- |
| Accuracy | | 0.6125 | 0.6587 * | 0.6513 |
| AUC | | 0.6534 | 0.7167 * | 0.7119 |
| Top Features | 1 | **Spike timing skewness (OC)** | **Spike timing skewness (OC)** | **KS statistic (OC)** |
|  | 2 | **Spike timing skewness (OI)** | **KS statistic (OC)** | **Spike timing skewness (OC)** |
|  | 3 | **KS statistic (OI)** | Mean FR in 100–250 ms (OC) | SD of spike timing (OC) |
|  | 4 | **KS statistic (OC)** | **Spike timing skewness (OI)** | Mean FR in 50–100 ms (OC) |
|  | 5 | Mean FR in 50–100 ms (OC) | SD of spike timing (OC) | **Spike timing skewness (OI)** |
|  | 6 | SD of spike timing (AI) | FRc index (AI) | Q1 spike timing (OC) |
|  | 7 | FRc index (AI) | SD of spike timing (AI) | Spike timing kurtosis (AC) |
|  | 8 | FRc index (OI) | **KS statistic (OI)** | **KS statistic (OI)** |
|  | 9 | SD of spike timing (AC) | Spike timing kurtosis (AC) | Q1 spike timing (OI) |

**Extended Data Figure 7-4.** This table summarizes the classification performance and the top-ranking features for the best model architecture (CatBoost) in the primary motor cortex (M1) across three independent training/testing repetitions (Repeat 0, 1, and 2). The best model architecture was determined based on the highest mean accuracy across repetitions (see Materials and Methods). For each repetition, the table lists the performance metrics (Accuracy and AUC) on the held-out test set, with the maximum values across repetitions indicated by asterisks (*). The top 9 features with the highest mean absolute SHAP values are listed in descending order of importance. Features that consistently ranked within the top 9 across all three repetitions are highlighted in bold text. Common features consistently identified include spike timing skewness (OC and OI) and KS statistic (OC and OI). Similar to M2, the classification accuracy in M1 is substantially lower than in the hippocampal and parahippocampal regions, consistent with its position at the lower end of the functional hierarchy.
