# Supplementary material for: Hierarchical Distribution of Reward Representation in the Cortical and Hippocampal Regions
Source: eNeuro. 2026 Feb 10;13(2):ENEURO.0256-25.2026. doi: 10.1523/ENEURO.0256-25.2026 (PMC12931971; doi:10.1523/ENEURO.0256-25.2026)
Supplement: Data 1 — The complete code, data, and computational environment required to reproduce the findings of this study are available at https://github.com/mokamotosan/soma_okamoto_lr_01_public.git. The repository contains a series of Jupyter Notebooks for analysis (/notebooks) and a Dockerfile to ensure a fully reproducible environment. The analysis is structured with individual notebooks for each of the six brain regions, which generate the results for Figs. 3-7 and Table 1. All analyses were performed within a Docker container based on a Python 3.10 image, utilizing key libraries such as PyCaret for modeling and SHAP for interpretation. To replicate all results, users should first build the Docker environment and then execute the Jupyter Notebooks in the sequence described in the repository's README.md file. Download Data 1, ZIP file. [file eneuro-13-ENEURO.0256-25.2026-s001.zip › soma_okamoto_lr_01_public-main/results/tuning/tuning_score_aggregated.pdf]

Table 1

Summary of Model Performance

			M1	M2	PPC	LEC	vCA1	dCA1
Sample size		Reward	915	1277	661	1298	1028	610
		Non-reward	891	1221	629	1164	750	518
		Total	1806	2498	1290	2462	1778	1128
Tuned models and performance on test data	CatBoost	Accuracy	0.6408	0.6524	0.6933	0.7704	0.8027	0.8083
		(SD)	(0.0248)	(0.0235)	(0.0403)	(0.0090)	(0.0416)	(0.0230)
		AUC	0.6940	0.7038	0.7597	0.8472	0.8828	0.8928
		(SD)	(0.0352)	(0.0234)	(0.0331)	(0.0085)	(0.0385)	(0.0217)
	LightGBM	Accuracy	0.6390	0.6569	0.6684	0.7618	0.8121	0.7935
		(SD)	(0.0102)	(0.0239)	(0.0368)	(0.0160)	(0.0319)	(0.0223)
		AUC	0.6824	0.7062	0.7386	0.8359	0.8809	0.8871
		(SD)	(0.0341)	(0.0280)	(0.0414)	(0.0206)	(0.0332)	(0.0180)
	XGBoost	Accuracy	0.6310	0.6369	0.6821	0.7573	0.7984	0.7994
		(SD)	(0.0394)	(0.0245)	(0.0506)	(0.0082)	(0.0309)	(0.0340)
		AUC	0.6792	0.6957	0.7440	0.8397	0.8787	0.8902
		(SD)	(0.0445)	(0.0190)	(0.0511)	(0.0106)	(0.0309)	(0.0196)

Table 1. Dataset statistics and classification performance of the hyperparameter-tuned models. The table summarizes the number of samples analyzed and the classification performance of the best-performing machine learning models (CatBoost, LightGBM: Light Gradient Boosting Machine, XGBoost: Extreme Gradient Boosting) selected for each brain region (M1, M2, PPC, LEC, vCA1, dCA1). Sample size (N): Represents the total number of valid neuron-condition data points used for classification (see Materials and Methods). Performance Metrics: Accuracy and Area Under the Curve (AUC) values represent the mean and standard deviation (SD) calculated across three independent repetitions of the hyperparameter tuning and evaluation process. Note that the hippocampal regions (dCA1, vCA1) consistently achieved the highest performance metrics with low variance, confirming the robustness of the functional hierarchy.
