# Supplementary material for: Hierarchical Distribution of Reward Representation in the Cortical and Hippocampal Regions
Source: eNeuro. 2026 Feb 10;13(2):ENEURO.0256-25.2026. doi: 10.1523/ENEURO.0256-25.2026 (PMC12931971; doi:10.1523/ENEURO.0256-25.2026)
Supplement: Data 1 — The complete code, data, and computational environment required to reproduce the findings of this study are available at https://github.com/mokamotosan/soma_okamoto_lr_01_public.git. The repository contains a series of Jupyter Notebooks for analysis (/notebooks) and a Dockerfile to ensure a fully reproducible environment. The analysis is structured with individual notebooks for each of the six brain regions, which generate the results for Figs. 3-7 and Table 1. All analyses were performed within a Docker container based on a Python 3.10 image, utilizing key libraries such as PyCaret for modeling and SHAP for interpretation. To replicate all results, users should first build the Docker environment and then execute the Jupyter Notebooks in the sequence described in the repository's README.md file. Download Data 1, ZIP file. [file eneuro-13-ENEURO.0256-25.2026-s001.zip › soma_okamoto_lr_01_public-main/results/tuning/tuning_score_aggregated_LEC.pdf]

Extended Data Figure 7-1

Model performance and top-contributing features across independent repetitions for LEC

Repeat		0	1	2
Accuracy		0.7808 *	0.7659	0.7645
AUC		0.8442 *	0.8568	0.8407
Top Features	1	ks-stat_actionI	post_outcome_ipsi_mean_spk/s_100_250ms	pre_action_ipsi_mean_spk/s_(-50)_0ms
	2	post_outcome_ipsi_mean_spk/s_50_100ms	ks-stat_actionI	ks-stat_actionI
	3	post_outcome_contra_quantile(25)	ks-stat_actionC	ks-stat_actionC
	4	ks-stat_actionC	ks-stat_outcomeI	**diff_per_sum_outcomeC**
	5	**diff_per_sum_outcomeC**	post_outcome_contra_quantile(25)	post_outcome_contra_quantile(75)
	6	pre_action_contralateral_std	post_outcome_contra_kurtosis	post_outcome_contra_skewness
	7	pre_action_ipsi_std	**diff_per_sum_outcomeC**	ks-stat_outcomeI
	8	post_outcome_ipsi_std	post_outcome_ipsi_std	pre_action_psi_std
	9	pre_action_ipsi_skewness	post_outcome_ipsi_median	pre_action_ipsi_median
