# Supplementary material for: Hierarchical Distribution of Reward Representation in the Cortical and Hippocampal Regions
Source: eNeuro. 2026 Feb 10;13(2):ENEURO.0256-25.2026. doi: 10.1523/ENEURO.0256-25.2026 (PMC12931971; doi:10.1523/ENEURO.0256-25.2026)
Supplement: Data 1 — The complete code, data, and computational environment required to reproduce the findings of this study are available at https://github.com/mokamotosan/soma_okamoto_lr_01_public.git. The repository contains a series of Jupyter Notebooks for analysis (/notebooks) and a Dockerfile to ensure a fully reproducible environment. The analysis is structured with individual notebooks for each of the six brain regions, which generate the results for Figs. 3-7 and Table 1. All analyses were performed within a Docker container based on a Python 3.10 image, utilizing key libraries such as PyCaret for modeling and SHAP for interpretation. To replicate all results, users should first build the Docker environment and then execute the Jupyter Notebooks in the sequence described in the repository's README.md file. Download Data 1, ZIP file. [file eneuro-13-ENEURO.0256-25.2026-s001.zip › soma_okamoto_lr_01_public-main/results/tuning/tuning_score_aggregated_vCA1.pdf]

Extended Data Figure 6-1

Model performance and top-contributing features across independent repetitions for vCA1

Repeat		0	1	2
Accuracy		0.8315 *	0.7753	0.8296
AUC		0.9022 *	0.8427	0.8977
Top Features	1	pre_action_contralateral_mean_spk/s_(-50)_0ms	pre_action_contralateral_mean_spk/s_(-100)_(-50)ms	pre_action_contralateral_mean_spk/s_(-100)_(-50)_ms
	2	ks-stat_actionI	ks-stat_actionI	ks-stat_actionC
	3	ks-stat_actionC	ks-stat_actionC	ks-stat_actionI
	4	post_outcome_contra_quantile(25)	diff_per_suum_ActionC	diff_per_sum_actionC
	5	pre_action_ipsi_std	pre_action_ipsi_std	**ks-stat_outcomeI**
	6	**ks-stat_outcomeI**	**ks-stat_outcomeI**	post_outcome_contra_quantile(25)
	7	diff_per_sum_outcomeC	ACG_and_FS_per_RS_ACGmed250	pre_action_ipsi_quantile(75)
	8	diff_per_sum_actionC	post_outcome_contra_quantile(75)	pre_action_ipsi_std
	9	ks-stat_outcomeC	post_outcome_ipsi_quantile(75)	ACG_and_FS_per_RS_ACGmed250
